# A teosinte-derived allele of ZmSC improves salt tolerance in maize

**DOI:** 10.3389/fpls.2024.1361422

**Published:** 2024-06-05

**Authors:** Xiaofeng Li, Qiangqiang Ma, Xingyu Wang, Yunfeng Zhong, Yibo Zhang, Ping Zhang, Yiyang Du, Hanyu Luo, Yu Chen, Xiangyuan Li, Yingzheng Li, Ruyu He, Yang Zhou, Yang Li, Mingjun Cheng, Jianmei He, Tingzhao Rong, Qilin Tang

**Affiliations:** ^1^ Maize Research Institute, Sichuan Agricultural University, Chengdu, China; ^2^ Agricultural Genomics Institute at Shenzhen, Chinese Academy of Agricultural Sciences, Shenzhen, China; ^3^ Pingliang Academy of Agricultural Sciences, Pingliang, China; ^4^ Animal Feeding and Management Department, Research Base of Giant Panda Breeding, Chengdu, China; ^5^ Horticulture Research Institute, Sichuan Academy of Agricultural Sciences, Chengdu, China; ^6^ School of Urban and Rural Planning and Construction, Mianyang Teachers’ College, Mianyang, China; ^7^ College of Grassland Resources, Southwest Minzu University, Chengdu, China

**Keywords:** salt stress, maize, wild relatives, transgenic *Arabidopsis*, CDPK

## Abstract

Maize, a salt-sensitive crop, frequently suffers severe yield losses due to soil salinization. Enhancing salt tolerance in maize is crucial for maintaining yield stability. To address this, we developed an introgression line (IL76) through introgressive hybridization between maize wild relatives *Zea perennis*, *Tripsacum dactyloides*, and inbred Zheng58, utilizing the tri-species hybrid MTP as a genetic bridge. Previously, genetic variation analysis identified a polymorphic marker on *Zm00001eb244520* (designated as ZmSC), which encodes a vesicle-sorting protein described as a salt-tolerant protein in the NCBI database. To characterize the identified polymorphic marker, we employed gene cloning and homologous cloning techniques. Gene cloning analysis revealed a non-synonymous mutation at the 1847th base of *ZmSC^IL76^*, where a guanine-to-cytosine substitution resulted in the mutation of serine to threonine at the 119th amino acid sequence (using *ZmSC^Z58^* as the reference sequence). Moreover, homologous cloning demonstrated that the variation site derived from *Z. perennis*. Functional analyses showed that transgenic *Arabidopsis* lines overexpressing *ZmSC^Z58^* exhibited significant reductions in leaf number, root length, and pod number, alongside suppression of the expression of genes in the SOS and CDPK pathways associated with Ca^2+^ signaling. Similarly, fission yeast strains expressing *ZmSC^Z58^* displayed inhibited growth. In contrast, the *ZmSC^IL76^* allele from *Z. perennis* alleviated these negative effects in both *Arabidopsis* and yeast, with the lines overexpressing *ZmSC^IL76^* exhibiting significantly higher abscisic acid (ABA) content compared to those overexpressing *ZmSC^Z58^*. Our findings suggest that ZmSC negatively regulates salt tolerance in maize by suppressing downstream gene expression associated with Ca2+ signaling in the CDPK and SOS pathways. The *ZmSC^IL76^* allele from *Z. perennis*, however, can mitigate this negative regulatory effect. These results provide valuable insights and genetic resources for future maize salt tolerance breeding programs.

## 1 Introduction

Salt stress is one of the major abiotic stresses, that significantly hinder agricultural production and limits the further improvement of crop yield (Zhang et al., 2023). In particular, maize is a glycophytic crop highly susceptible to salt stress. Moreover, most currently available maize germplasm resources lack salt-tolerant traits, and the underlying molecular mechanism of plant responses to salt stress remains unclear. However, the wild relatives of maize, *Zea perennis*, and *Tripsacum dactyloides*, retain beneficial traits that have been eliminated during the domestication process and represent a valuable gene pool for the genetic improvement of maize. Therefore, retrieving the lost alleles and strengthening research on the molecular mechanisms of salt stress response in maize are of great theoretical and practical significance.

Salt stress decreases soil hydraulic conductivity and increases the external osmotic pressure of plant roots (Pingle et al., 2022). Under salt stress, excessive ion uptake in plants leads to significant disruption of the dynamic ion and water balance, resulting in membrane damage and cell death (Zhu, 2016). Consequently, seed water absorption ability is significantly reduced, leading to greatly diminished germination rates, germination potential, and radicle development, making seedling establishment difficult. It has been reported that excessive uptake of Na^+^ in plants will compete with potassium ions, resulting in reduced K^+^ content, and the unbalanced Na^+^/K^+^ ratio leads to more severe damage (Rubio et al., 2020). Intensified salt stress can further cause oxidative stress, resulting in the accumulation of toxic compounds such as reactive oxygen species (ROSs). This can ultimately alter membrane functionality, impairing the cell’s ability to maintain proper ion and nutrient balances, and negatively affecting plant growth and development (Zhu, 2001; Yang and Guo, 2018).

To cope with salt stress, plants have developed various adaptive mechanisms (Yang et al., 2020). Organic osmoregulatory agents such as proline (Pro), soluble sugar, and glycine betaine (GB), play a pivotal role in preventing water loss in plants. Studies have shown a significant increase in the content of Pro and GB in maize under salt stress (Bano and Fatima, 2009). Additionally, the soluble sugar content of salt-tolerant maize lines has been reported to be higher than that of salt-sensitive lines. To enhance salt tolerance in maize, genes mediating shoot Na^+^ exclusion by withdrawing Na^+^ from the root xylem flow are known (Zhang et al., 2019, 2023).

Plant hormones play a pivotal role in the stress response, particularly abscisic acid (ABA), which is an endogenous signal molecule involved in regulating abiotic stresses in plants, such as salt stress (Sah et al., 2016; Li et al., 2024). When plants experience salt stress, the activation of the ABA signal pathway leads to the expression of downstream osmotic regulation response genes in *Arabidopsis* (Yu et al., 2020). Under salt stress, the osmotic potential and pre-dawn leaf water potential are reduced, leading to an increase in ABA levels, which consequently decreases the stomatal model parameters, thereby enhancing salt and drought resistance (Song et al., 2023). Furthermore, overexpression of *AtLOS5* promotes ABA biosynthesis in *Arabidopsis*, and the transgenic plant has shown stronger salt tolerance than the wild type (Zhang et al., 2016).

In response to salt stress, plants activate signaling transduction networks. Among these networks, the salt overly sensitive (SOS), calcium-dependent protein kinase (CDPK), and mitogen-activated protein kinase (MAPK) pathways play pivotal roles in transducing environmental cues perceived by plant cell membranes to target genes (Li and Nam, 2002). CDPKs are essential factors in abiotic stress tolerance, and CDPK regulates stress tolerance by modulating ABA signaling and reducing ROS accumulation (Asano et al., 2012). The SOS pathway plays a key role in regulating ion transport under salt stress (Zhou et al., 2022). In the SOS pathway, the cascade activation of *SOS3*, *SOS2*, and *SOS1* triggered by an increase in cellular Ca^2+^ concentration, activates *SOS1*, which promotes Na^+^ efflux, and overexpression of *SOS1* significantly enhanced the salt tolerance in *Arabidopsis* (Shi et al., 2003).

Iqbal et al. (2019) reported a tri-species hybrid called MTP (2n = 20M+ 34T + 20P = 74; M, T, and P, stand for autotetraploid maize, *T. dactyloides*, and *Z. perennis*, respectively). MTP has exhibited excellent salt tolerance, and its hybrids with maize are fertile, allowing for the development of a series of MTP-maize introgression lines with high salt tolerance utilizing MTP as the donor and maize inbred line as the recipient (Li et al., 2023). During genetic diversity analysis of MTP-maize introgression lines, a mutated gene *Zm00001eb244520* (designed as *ZmSC*) was detected in a salt-tolerant MTP-maize introgression line 76 (IL76), using its parent Zheng58 (Z58) as a reference. This gene was described as a salt-tolerant protein (Alexandrov et al., 2009), and a previous study showed that overexpression of *TaSC*, a homologous gene of *ZmSC*, could enhance *Arabidopsis* salt tolerance (Huang et al., 2012). In the current study, we aimed to elucidate the effect of the *ZmSC* on salt tolerance in maize under salt stress conditions and preliminarily unravel the regulatory mechanism of *ZmSC* in the salt response. To this end, we conducted experiments to determine the expression pattern of *ZmSC* under salt stress, investigate its molecular function, and identify the source of its variation. Collectively, our research provides valuable insights into the molecular mechanism of salt tolerance and provides genetic resources to facilitate the development of salt-tolerant maize varieties.

## 2 Materials and methods

### 2.1 Plant materials and growth conditions

In a previous study, a series of MTP-maize introgression lines were generated by utilizing the tri-species hybrid MTP as a donor and elite inbred line Zheng58 (Z58) as the acceptor through backcrossing and self-crossing, and IL76 (BC_9_F_5_) was one of the lines. To develop a near-isogenic line carrying the *ZmSC^IL76^* allele from IL76(BC_9_F_5_). the line was backcrossed to Z58 for three generations and then selfed to make it homozygous, resulting in the near-isogenic line NIL^IL76^ (BC_12_F_6_). In each generation, molecular markers were used to select for the introgressed *ZmSC^IL76^*, the molecular marker primers are listed in **Supplementary Table 3**. Field pollination was conducted in the experimental field of Sichuan Agriculture University (Chengdu, China) (30°26′N–31°26′N, 102°54′E–104°53′E). All the experiments of salt tolerance identification were performed in the greenhouse of Sichuan Agriculture University with 14 h of light at 28°C and 10 h of darkness at 23°C, and 75% humidity.

### 2.2 Cloning and bioinformatics analysis of the *ZmSC*

Genomic DNA and total RNA were isolated from inbred line Z58, IL76, wild relatives T. dactyloides, Z. perennis, and MTP. Electrophoresis on 1.5% agarose gels and sequencing were utilized to examine DNA and total RNA. The promoter and full-length *ZmSC* gene were amplified using DNA as a template, with primers designed online at NCBI (https://www.ncbi.nlm.nih.gov/tools/primer-blast/, accessed on 6 March 2023). Total RNA was reverse transcribed to cDNA, followed by amplification of CDS to detect sources of variation. The sequences were aligned using DNAMAN software. All primers were listed in **Supplementary Tables 3, 4**, the same below.

The Hidden Markov Model (HMM) profile of *ZmSC* domain UPF0220 downloaded from the Pfam database was employed to identify *ZmSC* genes in the maize genome (www.maizegdb.org, accessed on 6 March 2023), using the simple HMM search program of TBtools (Chen et al., 2020). To confirm the *ZmSC* domain, SMART and NCBI conserved Domain Data (CDD) search programs were utilized. ClustalW (Chenna et al., 2003) was utilized to carry out multiple sequence alignment analysis, and the phylogenetic tree was constructed by the neighbor-joining method of MEGA 7.0 with 1000 bootstrap replicates (Kumar et al., 2016).

### 2.3 *ZmSC* subcellular localization

The Vector pCAMBIA2300-Pro35s::eGFP and BM seamless cloning kit (Biomed) were used for the construction of recombinant vector pCAMBIA2300-Pro35s::ZmSC-eGFP. The recombinant vector was transformed into the *E.coli* DH5α competent cells using a heat shock protocol for propagation and validated by Sanger sequencing. Recombinant plasmids pCAMBIA2300-Pro35s::ZmSC-eGFP were transformed into the *Agrobacterium strain* GV3101 using the freeze-thaw method and infiltrated into four-week-old Tobacco (*Nicotiana. benthamiana*) leaves. For each transformation, three biological replicates were used. ER-mCherry and NLS-mCherry were used as the endoplasmic reticulum and nuclear marker, respectively. The signal was detected using a confocal microscope (Leica, Germany) 72 h after infiltration as described previously (Zou et al., 2018).

### 2.4 Transformation of *Arabidopsis* and fission yeast

The constructed vector pCAMBIA2300-Pro35s::ZmSC-eGFP was then transformed into the floral tissues of *Arabidopsis thaliana* via Agrobacterium-mediated floral dip method. Following transformation, plants were screened by PCR to confirm the presence of the *ZmSC* gene, resulting in developing three independent transgenic overexpression lines. From these lines, those exhibiting the highest *ZmSC* expression level, as determined by RT-qPCR, were subsequently selected for further experiments, including salt tolerance identification and physiological index measurement under NaCl stress.

Primers containing restriction sites for *SalI* and *BamHI* were used to amplify the target gene region. The digested products were recovered from the agarose gel using a DNA Recovery Kit (TIANGEN Biotech). Plasmid pREP1 was digested with restriction enzymes *SalI* and *BamHI* and connected with digested products using T4 DNA ligase. The method for transforming the recombinant vector into the *E.coli* DH5α competent cells, as well as validation, is the same as the method described in the 2.3 section. To transform the recombinant plasmid DNA into fission yeast (Schizosaccharomyces pombe *SPQ.01*), a mixture of 10 μl Carrier DNA, 1 μg recombinant plasmid DNA, 50 μl fission yeast competent cells, and 500 μl buffer was incubated in a water bath for 30 minutes in a 1.5 ml EP tube, followed by a 15-minute incubation at 42°C. The resulting product was centrifuged at 12,000 rpm for 15 seconds, and the supernatant was discarded. This process was repeated after adding 500 μl sterile ddH_2_O for washing. Reconstructed yeast was resuspended in 50 μl ddH_2_O and then daubed on MM medium. After 4–6 days of cultivation, the surviving single clones were picked up and expanded at 28°C, 200 rpm for 24h. After bacterial liquid PCR detection, the recombinant yeast was used for further identification of salt tolerance. For salt tolerance identification, positive yeast transformants and empty vector were both diluted to an OD600 of 0.8, and then further diluted to 10–^2^, 10–^3^, 10–^4^, and 10–^5^. Finally, 4 μl of the diluted cultures was cultured with MM medium containing NaCl.

### 2.5 RNA extraction and RT-qPCR

The total RNA of roots and leaves was extracted using the EasyPure Plant RNA Kit (TransGen Biotech, Beijing, China). RNA integrity was examined by electrophoresis in 1.5% agarose gel. Reverse transcription was immediately performed using the RevertAid First Strand cDNA Synthesis Kit. Primers were designed using an online tool from NCBI. RT-qPCR was conducted using the “SYBR” Premix Ex TaqTM kit (Takara, Japan) on a Roche LightCycler480 instrument. Glyceraldehyde 3-phosphate dehydrogenase (GAPDH) was used as an internal control. The relative abundance of transcripts was determined using the 2^-ΔΔCT^ method (Livak and Schmittgen, 2001).

We defined the relative expression as the ratio of expression levels in the experimental group (T) to that in the control group (CK). Here, T represents conditions of salt stress or the introgression line IL76, while CK represents normal conditions or inbred Z58. Relative expression was calculated using the formula:


$$Relative\;Expression\;=\;\frac{Expression\;Level\;\left(T\right)}{Control\;Group\;\left(CK\right)}$$

All experiments were performed three times to ensure accuracy.

### 2.6 Physiological indices measurements

Root and leaf samples were collected from 2-week-old seedlings under normal and 200 mM NaCl stress conditions for 7 d. the contents of Na^+^, K^+^, and Ca^2+^ were determined by atomic absorption spectrometry (Cushman et al., 2020). WinRhizo software (LC4800-II LA2400, Sainte Foy, Canada) was used to analyze the root traits, including total root length, volume, surface area, tip number, and average diameter.

The concentrations of phytohormones, including IAA (Indole acetic acid), GA (Gibberellic acid), JA (Jasmonic acid), ABA (Abscisic acid), and CTK (Cytokinin), were determined using Ruixinbio kits (Ruixin Biological Technology Co., Ltd, Quanzhou, China) by following manufacturer protocols. Each experimental procedure was conducted with three biological replicates.

### 2.7 Statistical analysis

Excel 2019 (Microsoft, Redmond, USA) and SPSS 27.0 (SPSS, Chicago USA) software were used for data analysis and collation, and OriginLab 2022 (Originlab, MA, USA) was used for plotting. We performed statistical analysis using t-tests and analysis of variance (ANOVA). All of the assays were performed in triplicated and repeated at least three times. Salt tolerance coefficients (STC) were calculated using the formula (Luo et al., 2017):


$$STC=\;\frac{Value\;under\;salt\;stress\;\left(T\right)}{Value\;under\;normal\;condition\;\left(CK\right)}\times 100\%$$

## 3 Results

### 3.1 Single nucleotide mutation leads to ZmSC protein variants

**Figure 1:**
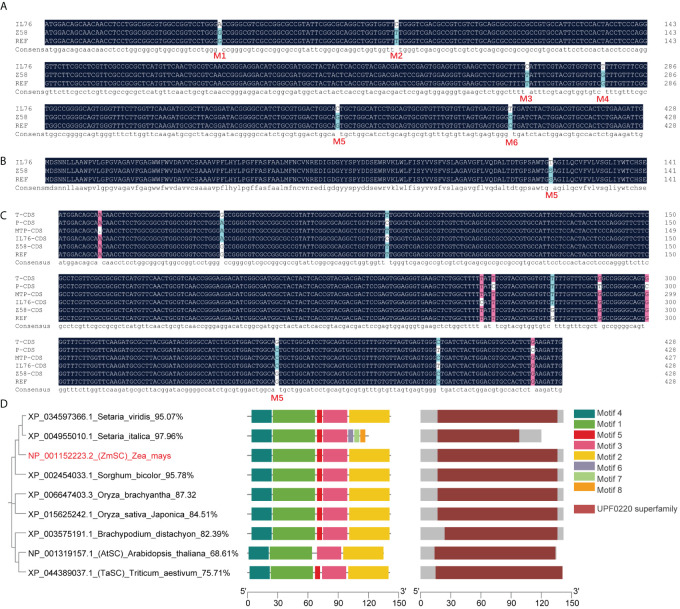
Mutation analysis of *ZmSC* coding region in IL76, and Phylogenetic tree and MEME analysis of ZmSC ortholog protein. **(A)** CDS sequence alignment result of *ZmSC* in the IL76, Z58, and B73 reference sequences; **(B)** The amino acid sequence alignment result of *ZmSC* in the IL76, Z58, and B73 reference sequences. **(C)** The results of homologous cloning of *ZmSC* CDS in wild parent *T. dactyloides* (T), *Z. perennis* (P), and MTP. **(D)** Phylogenetic tree, MEME analysis, and conserved domains of ZmSC ortholog protein.

According to the B73 reference genome (APG_V5), *ZmSC* comprises a full length of 2292 bp, consisting of four exons, and encodes a UPF0220 family protein comprising 142 amino acids. Sequence alignment revealed that the CDS of *ZmSC^Z58^* was consistent with that of the reference genome (APG_V5), and there were no mutations identified in the promoter region when comparing *ZmSC^Z58^* with *ZmSC^IL76^* (2 kb upstream of *ZmSC* of ATG). However, six SNP mutations (M1-M6) occurred in the CDS sequence of *ZmSC^IL76^* (**Figure 1A**), and M5 (G to C) at the 356th base resulting in an amino acid change from serine to threonine (S to T) distinguishing *ZmSC^Z58^* from *ZmSC^IL76^* (**Figure 1B**). Homologous sequence alignment showed that maize wild relatives *Zea perennis* and MTP carry the C allele at M5 but not in B73 and Z58 (**Figure 1C**). We conducted further analysis of the frequency of M5 in 36 maize genomes, including the NAM population, and 8 wild maize relatives (5 teosinte and 3 *Tripsacum dactyloides*) downloaded from the maize genome database. The results indicate that the frequency of M5 is 100% in the wild relatives but only 36% in the maize population (**Supplementary Figure 1**). Unfortunately, we were unable to collect these germplasm resources for salt tolerance identification, however, valuation of salt tolerance demonstrated that IL76 exhibits stronger salt tolerance compared to Z58 (**Supplementary Figures 2**, **3**). Additionally, IL76 exhibited resilience to drought stress (**Supplementary Figure 4**) and ABA stress (**Supplementary Figure 5**). These results suggest that M5 is an allele of *Zea perennis*, which is widely prevalent in wild maize relatives, and this allele may have been reintroduced to maize cultivation through genetic bridge MTP, and it could have been lost during maize domestication.

Based on the ortholog of amino acids, ortholog proteins of ZmSC were searched in OrthoDB (https://www.orthodb.org/, accessed on 8 March 2023) (**Figure 1D**), functional annotation revealed that these proteins are UPF0220 mainly transmembrane protein 50 homolog with a similar function, and five conservative motifs were detected using MEME program (https://meme-suite.org/meme/tools/meme, accessed on 8 March 2023) (**Figure 1D**). Among these, *TaSC* and *AtSC* have been shown to regulate salt tolerance in wheat (Huang et al., 2012). However, the function of *ZmSC* has not been reported. We further used the online tools (https://bioinformatics.psb.ugent.be/webtools/plantcare/html/, accessed on 8 March 2023) to predict the cis-element of 2 kb upstream of *ZmSC* of ATG, the promoter comprised a variety of functional elements. Including the most basic elements such as TATA-box and CAAT-box, as well as cis-element such as ABRE, AE-box, ARE, CAT-box, CGTCA-motif, G-Box, I-box, MBS, MYC, W-box, MYB, O2-site, TATC-box, TCA-element, TCCC-motif, TGACG-motif, as-1, and GC-motif, which are closely related to plant response to salt, drought, and other abiotic stress (**Supplementary Table 1**).

**Figure 2:**
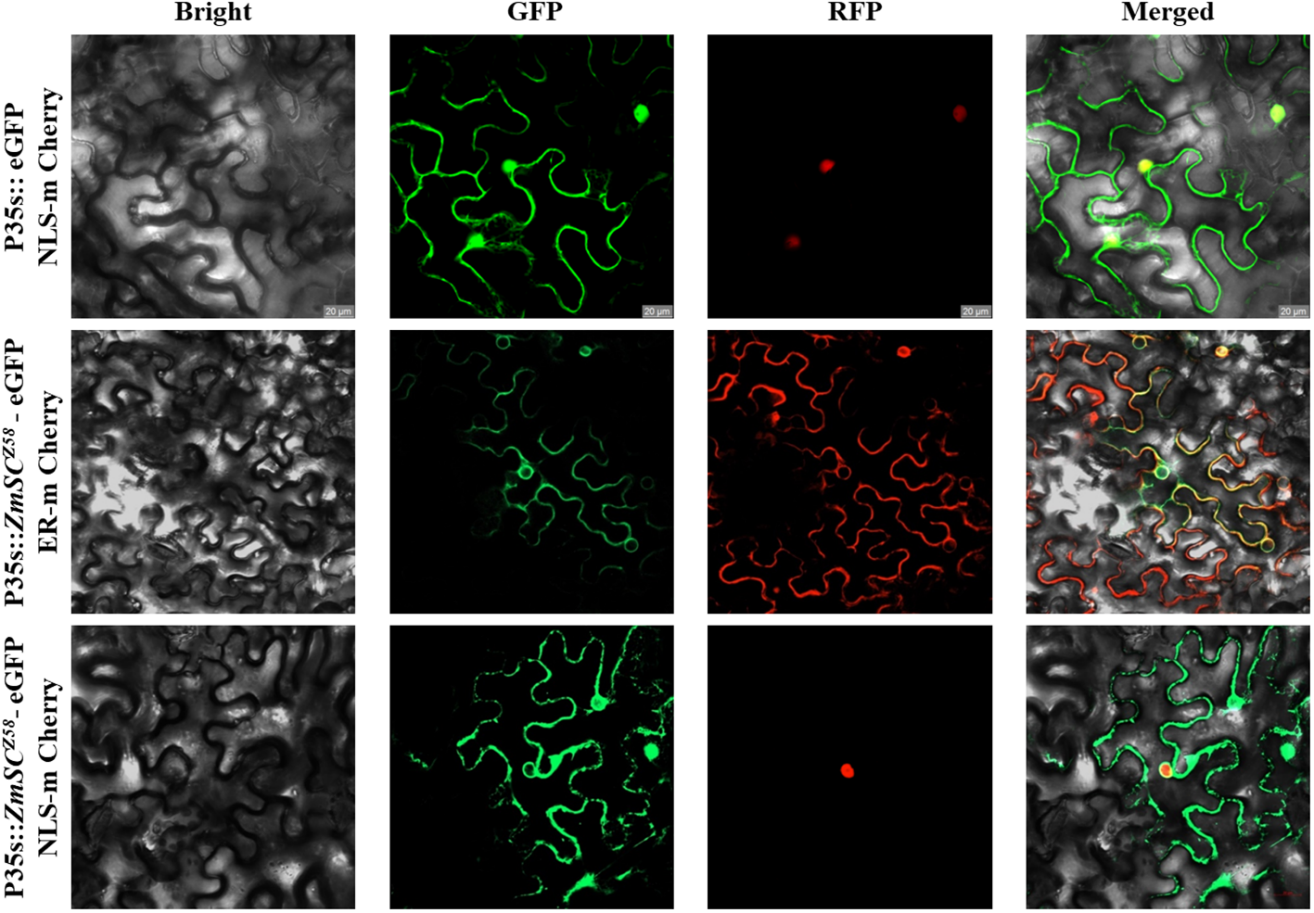
Subcellular localization of ZmSC-eGFP represents empty vector, NLS-mCherry (Nucleus marker chrrey), and ER-mChrrey (Endoplasmic reticulum marker chrrey) represents the nucleus and endoplasmic reticulum marker, respectively. Scale bar = 20 um.

To better understand *ZmSC*, we searched the *Zea mays* genome with the UPF0220 domain and validated it in the SMART database. Only one gene (*Zm00001eb100550*) belonging to the *ZmSC* family was identified at chromosome 2. This gene encodes a transmembrane protein 50A and is involved in late endosome to vacuole transport via multivesicular body sorting pathway. Currently, only one study has reported a potential association of this gene with maize grain weight (Zhou et al., 2020), and there is no research on its stress tolerance to date. To confirm the subcellular location of *ZmSC*, the *ZmSC* sequence was fused to green fluorescent protein (GFP) and transiently expressed in tobacco (*Nicotiana benthamiana*) epidermal cells, this revealed that the ZmSC-eGFP protein was primarily localized in the plasma membrane and nuclear membrane (**Figure 2**).

### 3.2 Downregulation of *ZmSC^IL76^* expression under salt stress

**Figure 3:**
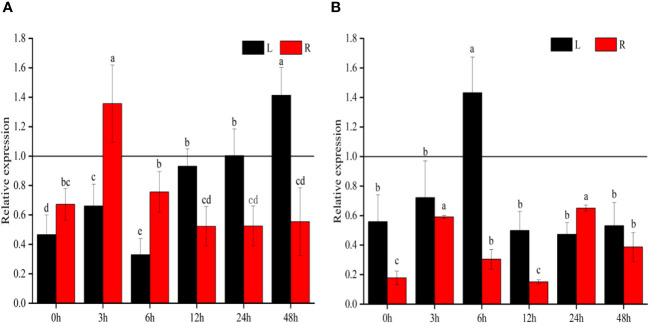
The relative expression of *ZmSC* at different time points under salt **(A)** and ABA **(B)** stress. L and R represent leaf and root tissues, respectively, the same as below. Different letters denote significant differences in the relative expression levels of *ZmSC* at different time points with the same tissues at the *P* < 0.05 level (n = 3; error bar = SD).

To investigate the expression patterns of *ZmSC*, inbred Z58, and IL76 seedlings (10 days old seedlings after germination) were exposed to salt stress (200 mM NaCl) and 100 μmol/L ABA stress for 48 hours. Leaf and root samples were collected at 0h, 3h, 6h, 9h, 12h, 24h, and 48-hour intervals for RT-qPCR analysis. The comparison of expression levels is conducted by evaluating the ratio of relative expression between *ZmSC^IL76^* and *ZmSC^Z58^*, represented as the expression level of *ZmSC^IL76^* to the expression level of *ZmSC^Z58^* at each time point (**Figure 3**). Our results showed that, under salt stress, except for the relative expression at 24h and 48h time points in leaf tissue and 3h time point at in root tissue, all other time points exhibited a ratio less than 1.0, indicating significant differences in the response of *ZmSC^IL76^* and *ZmSC^Z58^* to salt stress and the expression of *ZmSC^IL76^* was suppressed (**Figure 3A**). Under ABA stress conditions, the expression of *ZmSC^IL76^* appears to be suppressed, similar to the situation under salt stress (**Figure 3B**). These results indicate that compared to *ZmSC^Z58^*, *ZmSC^IL76^* exhibits lower expression levels under salt and ABA stress at most time potions.

### 3.3 *ZmSC* negatively regulated salt tolerance

**Figure 4:**
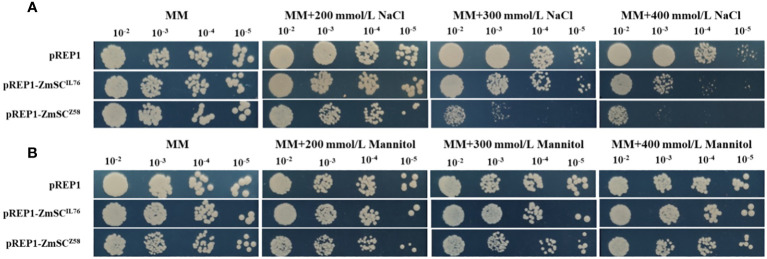
Expression of fission yeast transformants on different concentrations of NaCl **(A)** and Mannitol **(B)** MM medium.

To investigate the role of *ZmSC* in stress responses, we constructed the expression vector pREP1-ZmSC^Z58^ and pREP1-ZmSC^IL76^ in fission yeast, while the empty vector pREP1 served as the control. All strains grew normally on MM medium; however, their growth on MM medium supplemented with gradient NaCl was inhibited to varying degrees. Notably, the growth of pREP1-ZmSC^Z58^ strain was severely affected when the NaCl concentration was above 300 mM, followed by pREP1-ZmSC^IL76^, while pREP1 exhibited the least inhibitory effect (**Figure 4A**). Under mannitol stress, no significant difference was observed among the three strains (**Figure 4B**). These results indicate that *ZmSC^Z58^* and *ZmSC^IL76^* play a negative regulatory role in the salt tolerance of yeast, with *ZmSC^Z58^* exhibiting a stronger negative effect.

**Figure 5:**
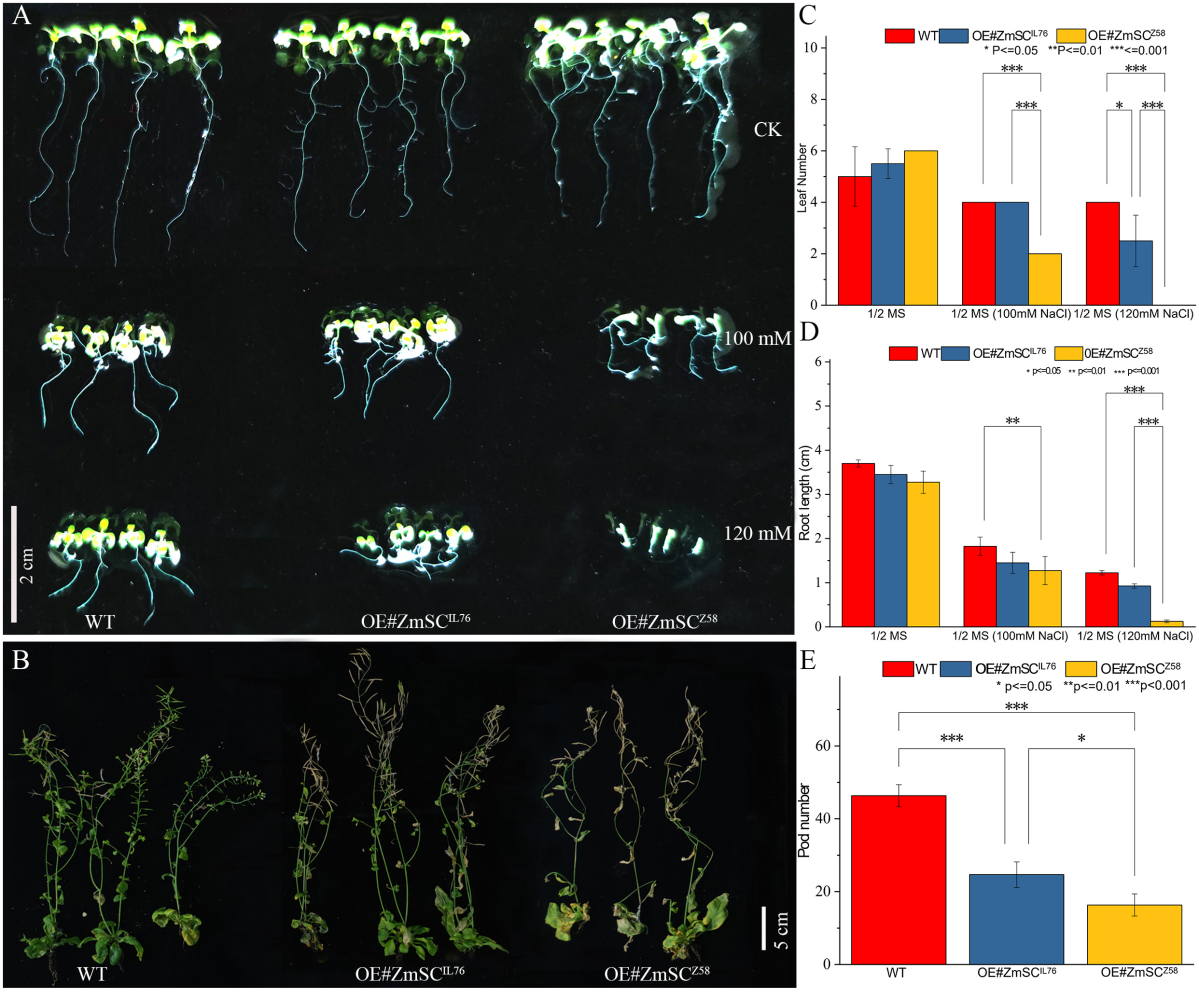
Phenotypes and indicators of wild-type (WT) and *ZmSC-*overexpressing (OE) *Arabidopsis* lines under stress conditions. **(A)** Growth of WT and OE seedlings under different stress conditions: 1/2 MS medium (control, CK), 1/2 MS supplemented with 100 mM NaCl, and 120 mM NaCl, Scale bar = 2 cm. Comparative analysis of leaf number and root length is presented in **(C)** and **(D)**, respectively. **(B)** Growth of three-week-old WT and OE plants after four weeks of treatment with 150 mM NaCl. Scale bar = 5 cm. Comparative analysis of pods is shown in **(E)**. Asterisks (*, **, and ***) indicate significant differences at *P* < 0.05, *P* < 0.01, and *P* < 0.001 levels, respectively, (n = 3; error bars = SD).

To investigate the role of *ZmSC^Z5^* ^8^ and *ZmSC^IL76^* in salt stress responses in detail, Two overexpression vectors, pCAMBIA2300-Pro35_S_::ZmSC^Z58^-eGFP and pCAMBIA2300-Pro35_S_::ZmSC^IL76^-eGFP, were constructed. Subsequently, they were transferred into *Arabidopsis thaliana* (WT). As a result, homozygous T3 lines were developed, and lines OE-IL76–1 (OE#ZmSC^IL76^) and OE-Z58–13 (OE#ZmSC^Z58^) showed a high expression level of *ZmSC* (**Supplementary Figure 6**) compared with WT. Therefore, these two lines were selected for identification of salt tolerance, while the WT was used as a control. After 2 weeks of growth, there were no significant differences among the three plants under normal conditions (1/2 MS medium). However, under salt stress conditions, there were noticeable differences, with OE#ZmSC^Z58^ showing more pronounced salt damage at both the seeding stage (**Figure 5A**) and mature stage (**Figure 5B**) than OE#ZmSC^IL76^. Comparative analysis of leaf number (**Figure 5C**), root length (**Figure 5D**), and the number of mature pods (**Figures 5B, E**) has all confirmed this phenomenon. Salt tolerance analysis of the near-isogenic lines NIL^IL76^ (BC_12_F_6_), which were constructed through backcrossing with Z58 and self-crossing (see methods 2.1), also showed that NIL^IL76^ had a stronger growth trend compared to Z58 (**Supplementary Figure 7**). Based on these results, it appears that *ZmSC* has a negative impact on the early growth and development of plants under salt stress, while the *ZmSC* mutation appears to alleviates this negative regulatory impact.

### 3.4 *ZmSC^IL76^* promotes ABA accumulation

**Figure 6:**
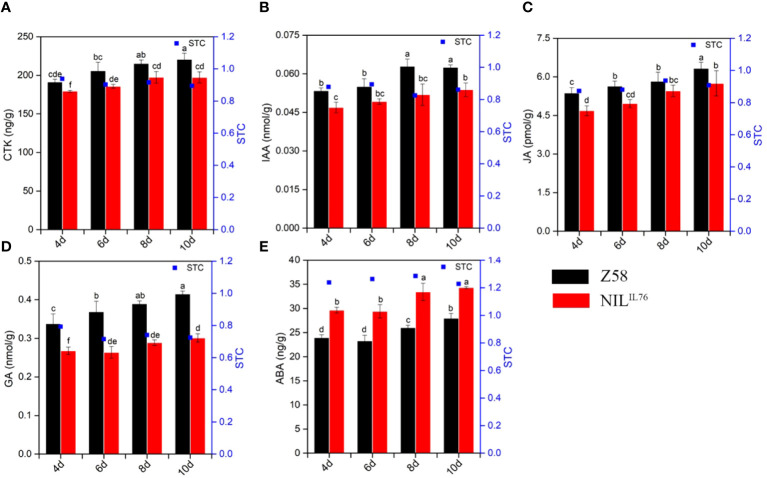
Phytohormone content dynamic changes under salt stress of inbred line Z58 and NIL^IL76^. **(A–E)** represents the GA, ABA, JA, IAA, and CTK phytohormone content, respectively. Different letters indicate significant differences at the *P* < 0.05 level, the same as below.

To determine whether phytohormones are involved in the regulation of *ZmSC*-mediated salt tolerance, we analyzed the level of five levels in Z58 and NIL^IL76^ under salt stress, including GA, ABA, JA, IAA, and CTK. First, NIL^IL76^ was germinated under normal and 200 mM NaCl stress, with phytohormone content measured on days 4, 6, 8, and 10, respectively, with Z58 serving as a control. The result showed that the hormone content of both NIL^IL76^ and Z58 increased to varying degrees as the duration of salt stress increased. Phytohormones such as CTK, IAA, JA, and GA in NIL^IL76^ were significantly lower than Z58 under salt stress, except for ABA, which was higher in NIL^IL76^ than in Z58 under salt stress (**Figure 6**). And the same result was observed in the ABA content determination assays of overexpression *Arabidopsis*, with OE#ZmSC^IL76^ showing significantly higher levels than OE#ZmSC^Z58^ under salt stress (**Supplementary Figure 10**). This indicates that *ZmSC^IL76^* may enhance the accumulation of ABA to response the salt stress.

### 3.5 The upstream transcription factor of *ZmSC* is associated with stress resistance

To explore the potential molecular regulatory mechanisms of upstream transcription factors of *ZmSC*, we selected the 2 kb sequence upstream of the ATG of ZmSC as the promoter sequence. Using the online tool (http://planttfdb.gao-lab.org/, accessed on 12 October 2022), we predicted 24 putative transcription factors belonging to various gene families, including NAC (11), bZIP (3), TCP (2), BBR-BPC (2), LBD (1), WOX (1), G2-like (1), C2C2-Dof (1), GRAS (1) and MADS (1) (**Supplementary Table 2**). From the 24 candidate transcription factors, we successfully cloned seven closely associated with abiotic stress responses for subsequent Yeast one-hybrid assay (Y1H), including *ZmNAC-tf79*, *ZmBBR-BPC-tf4*, *ZmC2C2-Dof-tf29*, *ZmBBR-BPC-tf3*, *ZmMADS-tf1*, *ZmNAC-tf112* and *ZmbZIP-obf1*.

**Figure 7:**
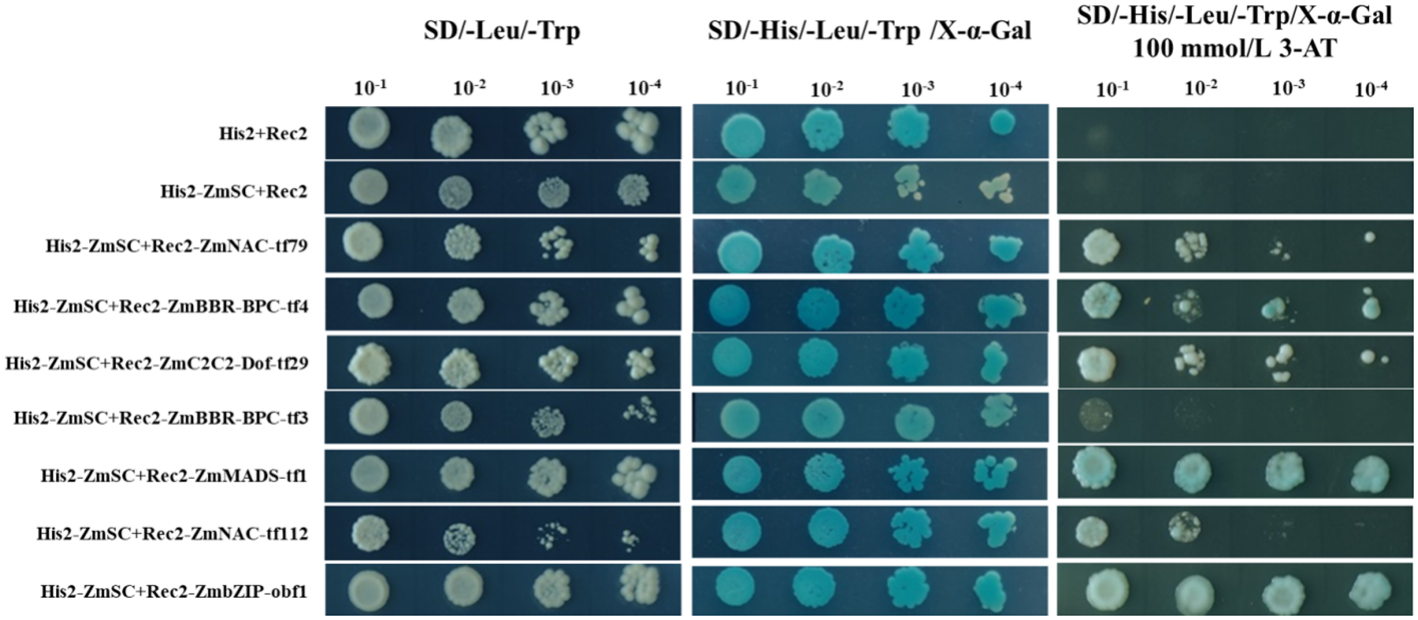
Yeast one-hybrid assays of upstream transcription factors of *ZmSC*.

The promoter of *ZmSC* was inserted into the pHis2 vector, whereas the CDS of the candidate transcription factors was inserted into the pGADT7-Rec2 vector. The recombinant plasmids His-ZmSC+Rec2-target_gene were independently introduced into the genome of the AH109 yeast strain, with the empty vectors His2+Rec2 and His2-ZmSC+Rec2 serving as control. All yeast cells were grown successfully on SD/-Leu/-Trip medium, except for His2-ZmSC+Rec2-ZmNAC-tf112, whose growth was inhibited when diluted 1000-fold. For all yeast cells transformed with target genes, growth was observed when placed on the SD/-His/-Leu/-Trip/X-α-Gal medium. When grown in the presence of 100 mM 3-AT on the SD/-His/-Leu/-Trip/X-α-Gal medium, all yeasts grew normally at a concentration of 10–^1^ except for the control and His2-ZmSC+Rec2-ZmBBR-BPC-tf3 which failed to grow. Therefore, we conclude that all transcription factors, except *ZmBBR-BPC-tf3*, interacted with *ZmSC* (**Figure 7**). The RT-qPCR assays confirmed that all six transcription factors, except for *ZmBBR-BPC-tf4*, exhibited a significant response to salt stress (**Supplementary Figure 8**). However, transcription activation assays indicated that *ZmNAC-tf79*, *ZmNAC-tf112*, and *ZmbZIP-obf1* possessed transcriptional activation ability (**Supplementary Figure 9**).

### 3.6 Overexpression of *ZmSC* in *Arabidopsis* suppressed the expression of the downstream genes in SOS and CDPK pathways

**Figure 8:**
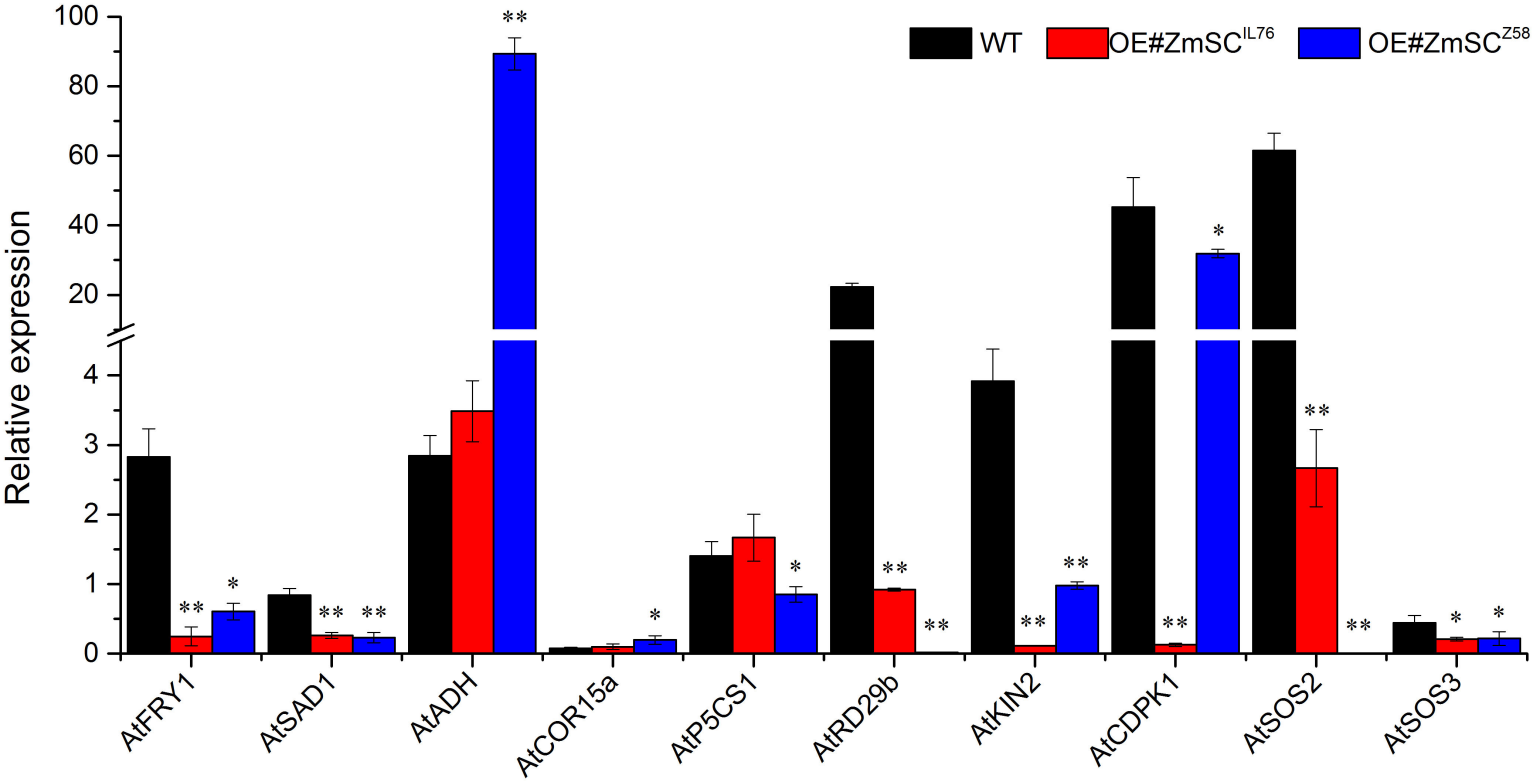
Expression of salt tolerance-related genes in *Arabidopsis thaliana* overexpressed by *ZmSC* under salt stress. The six-week-old wild type (WT), OE#ZmSC^Z58^, and OE#ZmSC^IL76^ plants were exposed to normal and 150 mM NaCl stress for 5 days. Leaf samples were collected for RT-qPCR analysis. The graph presents the mean ± standard deviation of three biological replicates. Asterisks (* and **) indicate significant differences at the *P* < 0.05, and *P* < 0.01 levels, respectively.

In a previous study, it was demonstrated that the overexpression of *TaSC*, a homolog of *ZmSC*, significantly affected the expression of a series of genes involved in CDPK and SOS pathways in *Arabidopsis* (Huang et al., 2012). In this study, we selected ten genes involved in CDPK and SOS pathway to investigate their response to salt stress in leaves of transgenic *Arabidopsis* lines overexpressing *ZmSC^Z58^* and *ZmSC^IL76^*, with wild-type *Arabidopsis* used as control (**Figure 8**). To characterize the changes in gene expression under salt stress (150 mM NaCl) compared with normal condition (ddH_2_O, CK), we defined the ratio of expression levels under stress conditions (T) to those under control conditions (CK) as the relative expression level (T/CK), The results showed that in the WT line, the expression, of seven genes (*AtFRY1*, *AtADH*, *AtP5CS1*, *AtRD29b*, *AtKIN2*, *AtCDPK1*, *AtSOS2*) were up-regulated under salt stress compared to normal condition (relative expression > 1). However, the most of gene expression in OE#ZmSC^Z58^ and OE#ZmSC^IL76^ were down-regulated and exhibited a consistent trend (relative expression level < 1), such as *AtFRY1*, *AtSAD1*, *ATCOR15a*, *AtRD29b*, *AtKIN2* and *AtSOS3*. Interestingly, under salt stress, the expression of genes *AtP5CS1* and *AtSOS2* appeared to be up-regulated in OE#ZmSC^IL76^, while being down-regulated in OE#ZmSC^Z58^. These results suggest that overexpression of *ZmSC^Z58^* and *ZmSC^IL76^* in *Arabidopsis* inhibits the expression of these genes in both the CDPK and SOS pathways.

## 4 Discussion

### 4.1 MTP is a genetic bridge to retrieve superior alleles from wild species *Zea perennis* and *Tripsacum dactyloides*

Crop wild relatives (CWRs) are a valuable reservoir of genetic resources that can be used to improve the genetic traits of cultivated species. Several studies have demonstrated the potential of maize wild relatives to enhance plant architecture (Tian et al., 2019), resist gray leaf spot (Zhang et al., 2017), increase protein content (Huang et al., 2022), improve yield (Wang et al., 2022), and enhance drought (Kumar et al., 2022). However, progress has been limited in improving salt tolerance in maize using wild species, likely due to the potential salt-tolerant wild relatives of maize being polyploid, such as *T. dactyloides* and *Z. perennis*. Higher ploidy levels may result in barriers that impede the genetic exchange with maize. Fortunately, recent work by Iqbal et al. (2019) and Yan et al. (2020) have reported a new tri-species hybrid MTP, which possesses the genomes of *Z. mays*, *T. dactyloides*, and *Z. perennis*. Li et al. (2023) confirmed that MTP exhibited strong salt tolerance, and a series of salt tolerance introgression lines have been screened from the backcross progeny of MTP with maize, using MTP as a genetic bridge. Cold tolerance introgression lines (He et al., 2023) and novel allotetraploid maize (Iqbal et al., 2023) have also been created. The introgression line IL76 in this study is one of those introgression lines. Sequence alignment analysis of the homologs revealed that the mutant site of *ZmSC^IL76^* leads to amino-acid changes present in both *Z. perennis* and MTP (**Figure 1C**), highlighting the potential of MTP as a gene pool for retrieving superior alleles from wild relatives.

### 4.2 *ZmSC* negatively regulates salt stress

We expressed *ZmSC* in both *Arabidopsis* and yeast and observed no significant differences in plant and yeast strain growth under normal conditions, suggesting that neither *ZmSC^Z58^* nor *ZmSC^IL76^* play a significant role in plant development under normal conditions. However, under salt stress, overexpression of *ZmSC^Z58^* and *ZmSC^IL76^* in *Arabidopsis* and yeast resulted in significant inhibition compared to WT (**Figures 4**, **5**). Notably, *ZmSC^Z58^* transgenic plants and yeasts display even greater salt sensitivity than *ZmSC^IL76^* transgenic plants and yeasts (**Figures 4**, **5**). These results suggest that *ZmSC^Z58^* acts as a negative regulator of salt tolerance in both *Arabidopsis* and yeast and that this negative regulatory effect is impeded when the gene is mutated. This conclusion was further supported by salt stress experiments conducted on NIL^IL76^ (**Supplementary Figure 7**). The intriguing aspect is the contrast between our findings, where overexpression of ZmSC negatively regulates salt tolerance in *Arabidopsis*, and the previous research indicating that the overexpression of the homologous gene *TaSC* had a positive impact on salt tolerance in *Arabidopsis* (Huang et al., 2012). This contrasting phenomenon may be explained by differences in the interaction of overexpressed genes. In our study, overexpression of *ZmSC* appears to inhibit the expression of *AtFRY1*, while overexpression of *TaSC* promotes the expression of *AtFRY1.* Plant domestication and selection are closely linked to environmental adaptations. Cao et al. (2019) reported that an allele conferring an amino acid variant in *ZmHKT2* enhances maize salt tolerance and likely underwent positive selection during maize domestication. Similarly, the allele found in *Z. perennis* in our study suggests that this variant may have experienced negative selection during maize domestication, leading to its loss or reduced prevalence in the maize population (**Supplementary Figure 1**).

### 4.3 *ZmSC* participates in salt stress response via ABA pathway

Abscisic acid (ABA) is a crucial hormone involved in stress response, including salt stress. Previous studies in maize have established that salt stress induces ABA synthesis, leading to the activation of the ABA signaling pathway. This activation, in turn, regulates the expression of downstream osmotic stress-related genes, thereby orchestrating the plant’s response to stress (Bahrun et al., 2002; Sah et al., 2016). In our study, we conducted Y1H assays and identified significant interactions between gene *ZmSC* and key transcription factors, namely *ZmNAC-tf79*, *ZmNAC-tf112*, and *ZmbZIP-obf1* (**Figure 7**). Importantly, the binding region of these transcription factors contained cis-element such as ABRE, CGTCA-motif, and TGACG-motif (**Supplementary Table 1**). Notably, ABRE has been previously demonstrated to play a crucial role in the ABA response (Finkelstein et al., 2005), and previous research has established that these three genes were associated with plant drought stress (Wang et al., 2020a; Cao et al. (2019)). Our RT-qPCR assays revealed that these transcription factors were induced by NaCl stress (**Supplementary Figure 8**). Concurrently, we observed a significantly higher accumulation of ABA in NIL^IL76^ (**Figure 6**) and OE#ZmSC^IL76^ (**Supplementary Figure 10**) compared to inbred Z58 and OE#ZmSC^Z58^ under salt stress conditions. *ZmSC* significantly responds to ABA stress and shows the different expression patterns between Z58 and IL76 (**Figure 3B**). These findings substantiate the potential association between ZmSC and the ABA pathway in the modulation of salt tolerance.

Moreover, recent research has highlighted the role of Sorting Nexin 2 proteins in modulating the trafficking and protein levels of the ABA exporter ABCG25, impacting cellular ABA levels (Liang et al., 2022), and multivesicular body pathway modulate the turnover and activity of ABA receptors and downstream regulators (Wang et al., 2020b). Given that *ZmSC* encodes for a transmembrane 50A-like protein involved in late endosome to vacuole transport via multivesicular body sorting pathway, suggesting a potential essential role for *ZmSC* in regulating salt tolerance by modulating the levels of ABA. Compared to ZmSC^Z58^, the mutation in ZmSC^IL76^ may result in reduced ABA transport, decreased ABA degradation, and regulated salt tolerance.

### 4.4 *ZmSC* is involved in the Ca^2+^-mediated response of salt stress

Ca^2+^ serves the function as a second messenger by interacting with Ca^2+^-sensing domains of CDPK (Wan et al., 2007). Cellular exposure to salt and other stresses increases Ca^2+^ levels, activating the CDPK and SOS signaling pathway to regulate downstream gene expression in response to stress (Shi et al., 2000; Ludwig et al., 2004). In a previous study, overexpression of *TaSC* enhanced the salt tolerance and increased Ca^2+^ content in *Arabidopsis*, likely due to the Ca^2+^ accumulation in transgenic *Arabidopsis* under salt stress, further activates CDPK pathway gene expression (Huang et al., 2012). The lack of activation in plasma membrane ion-binding channel activity, leading to a failure to release Ca^2+^ (Edel and Kudla, 2016), results in the down-regulation of genes, including downstream genes in the CDPK and SOS pathways.

In our study, under salt stress, the Ca^2+^ content significantly decreased in both IL76 and Z58, with IL76 exhibiting a higher level compared to Z58 (**Supplementary Figure 3E**). RT-qPCR assays revealed that the expression of genes *AtFRY1*, *AtSAD1*, *AtRD29b*, *AtKIN2*, *AtCDPK1* in the CDPK pathway and *AtSOS2*, *AtSOS3* in the SOS pathway was significantly down-regulated in overexpression *ZmSC^Z58^* and *ZmSC^IL76^ Arabidopsis* compared with WT (**Figure 8**). This finding contrasts with the result reported by Huang et al. (2012), suggesting that the overexpression of *ZmSC^Z58^* and *ZmSC^IL76^* suppresses the expression of downstream salt tolerance-related genes in the CDPK and SOS pathways in *Arabidopsis*.

**Figure 9:**
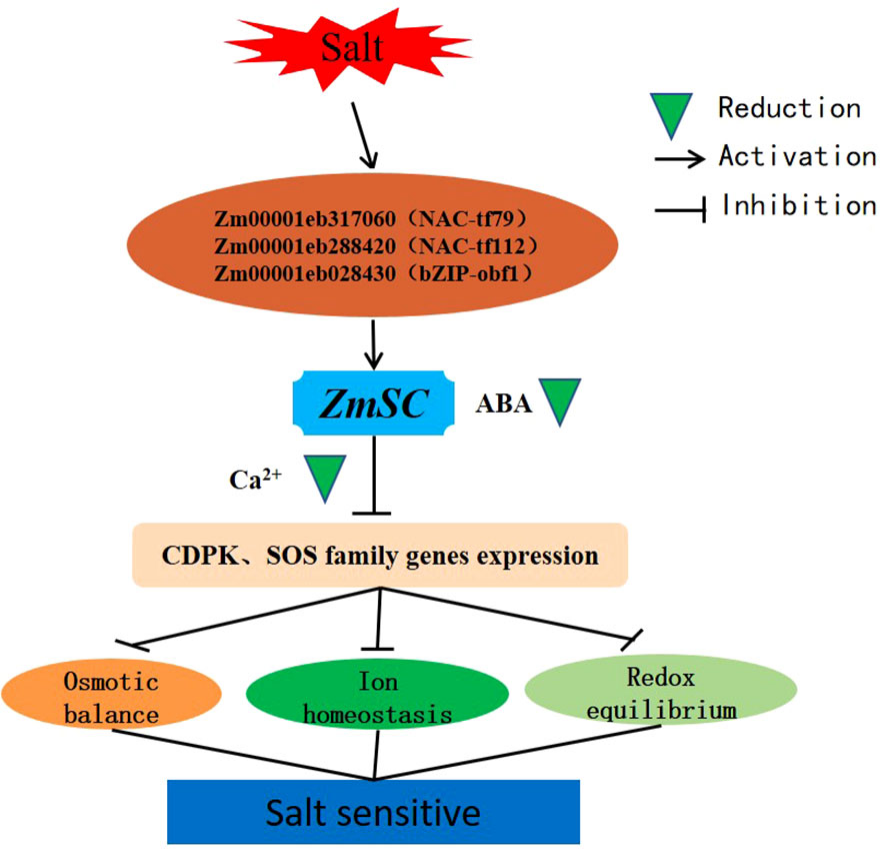
Working model of *ZmSC* in response to salt stress.

Given that, we speculate that *ZmSC* expression is activated upon recognition of the ABA signal, followed by transport of ABA to the vacuole through the *ZmSC*-mediated vacuolar sorting pathway, leading to its degradation. Ultimately, gene function in the Ca^2+^-mediated CDPK and SOS pathways becomes inhibited, leading to the disruption of ion homeostasis, osmotic balance, and oxidative regulation, consequently negatively regulating salt tolerance in plants (**Figure 9**).

## 5 Conclusion

In conclusion, our study demonstrated that *ZmSC* is expressed under salt and ABA stresses, functioning as a negative regulator of salt stress by suppressing the activation of genes in CDPK and SOS pathways under salt stress. Additionally, we observed that an allele derived from *Z. perennis* can alleviate this negative regulatory effect and enhance salt tolerance in maize. Therefore, our research establishes a robust foundation and provides valuable material for the molecular design breeding for salt tolerance in maize.

## Data availability statement

The original contributions presented in the study are included in the article/**Supplementary Material**. Further inquiries can be directed to the corresponding author.

## Author contributions

XFL: Conceptualization, Writing – original draft, Writing – review & editing, Data curation, Investigation, Visualization. QQM: Conceptualization, Data curation, Software, Visualization, Writing – original draft, Writing – review & editing. XYW: Data curation, Investigation, Writing – review & editing. YFZ: Investigation, Writing – review & editing. YBZ: Writing – review & editing. PZ: Writing – review & editing. YYD: Writing – review & editing. HYL: Writing – review & editing. YC: Writing – review & editing, Investigation. XYL: Writing – review & editing. YZL: Writing – review & editing. RYH: Writing – review & editing. YZ: Writing – review & editing, Investigation. YL: Writing – review & editing. MJC: Writing – review & editing. JMH: Writing – review & editing, Supervision. TZR: Writing – review & editing, Conceptualization. QLT: Writing – review & editing, Conceptualization, Funding acquisition, Project administration, Supervision, Writing – original draft.
