# Supplementary material for: A teosinte-derived allele of ZmSC improves salt tolerance in maize
Source: Front Plant Sci. 2024 Jun 5;15:1361422. doi: 10.3389/fpls.2024.1361422 (PMC11188391; doi:10.3389/fpls.2024.1361422)
Supplement: Supplementary file 1 [file DataSheet_1.docx]

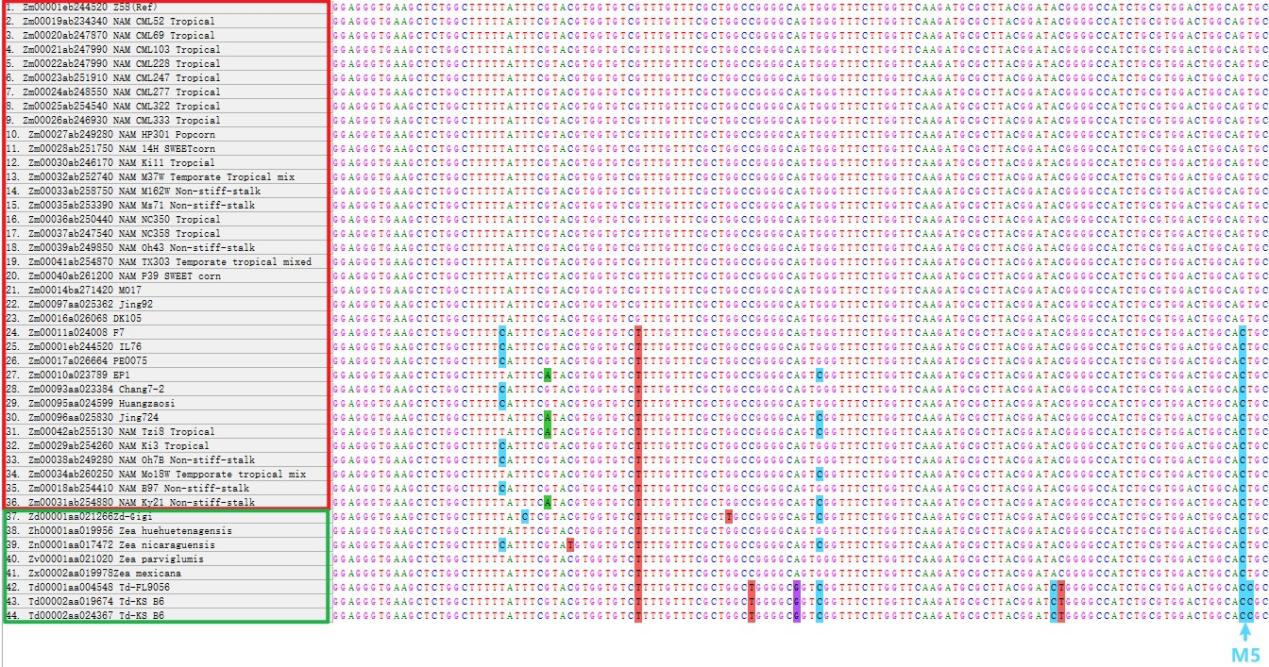


Supplementary Fig. 1 Homologous alignment of *ZmSC* in maize and wild relatives. The red box indicates cultivated maize lines, and the green box represents wild relatives.


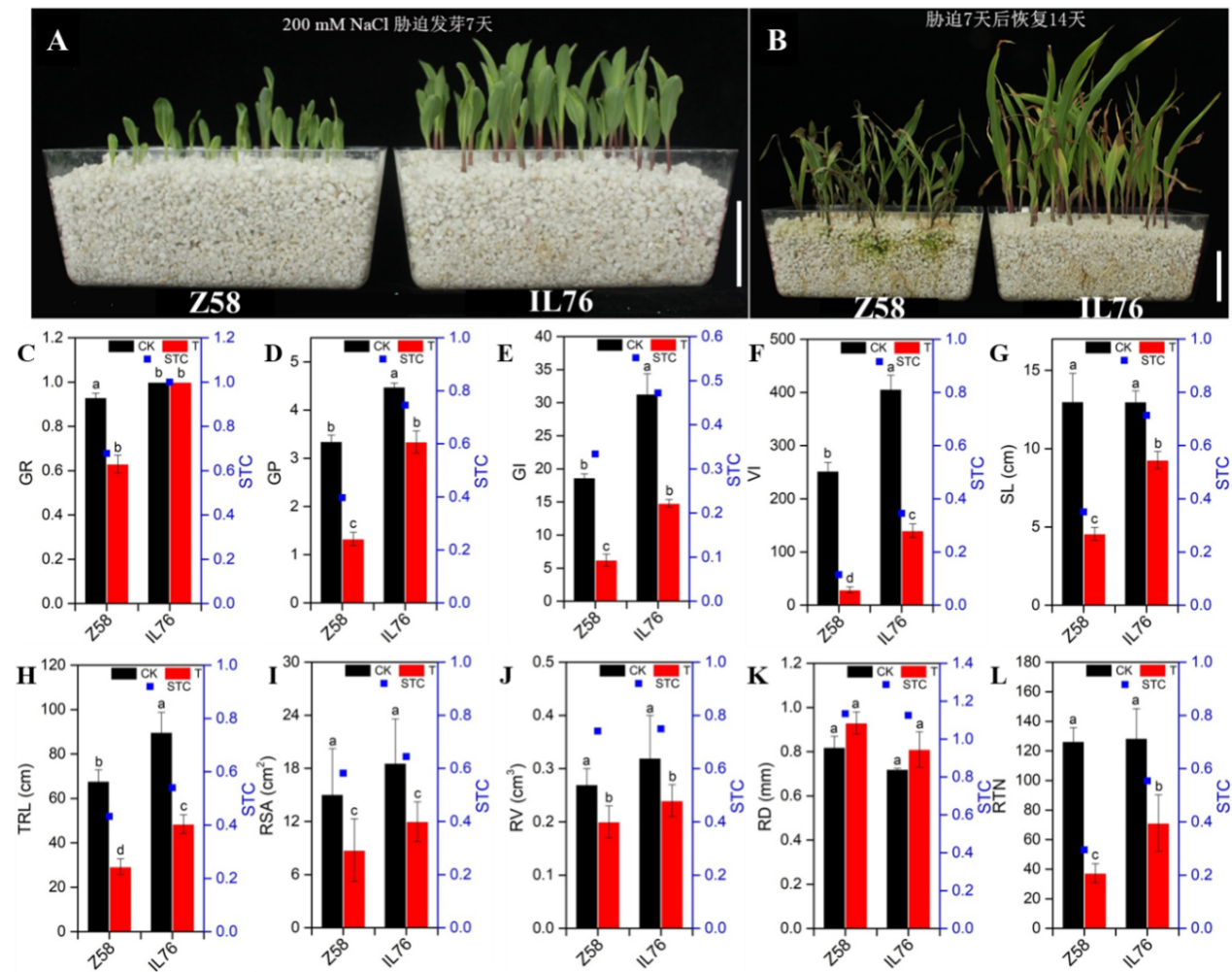


Supplementary Fig. 2 Phenotypic responses of IL76 and Z58 under normal (CK) and 200 mM salt stress (T) at the germination stage. A shows the plant after 7 days of salt stress. B shows the plant after 7 days of salt stress. C-L show the germination rate (GR), germination potential (GP), germination index (GI), vigor index (VI), shoot length (SL), total root length (TRL), root surface area (RSA), root volume (RV), root diameter (RD), and root tip number (RTN) after 7 days of salt stress, respectively. STC is the salt tolerance coefficient, the same as below. The data in the figure are the mean ± standard deviation of 5 biological replicates. The bars in the A and B represent 5 cm. Different lowercase letters indicate significance at the *P* < 0.05 level, the same as below.


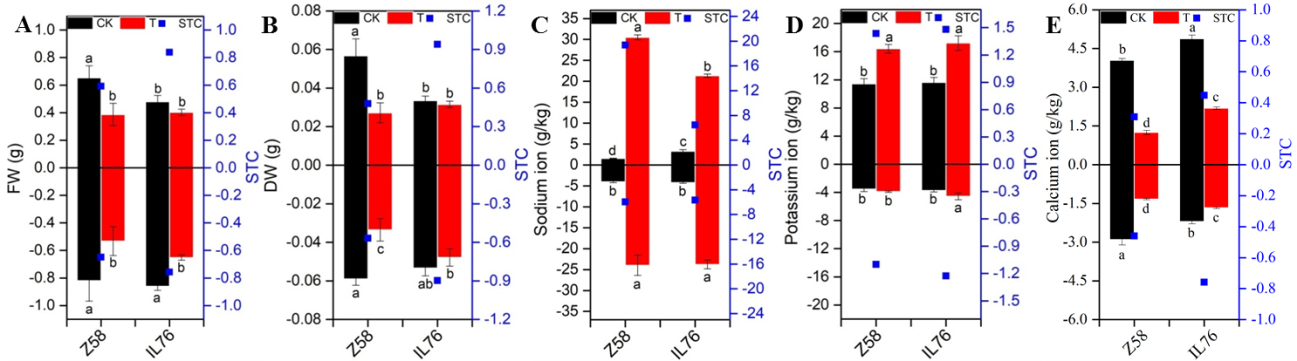


Supplementary Fig. 3 Physiological indices of IL76 and Z58 under normal (CK) and 200 mM salt stress (T) for 10 days at the germination stage. A and B show FW (fresh weight) and DW (dry weight), respectively. C-E show the content of Sodium, potassium, and Calcium ion. Positive values correspond to the leaf physiological indices, while negative value denote root physiological indices.


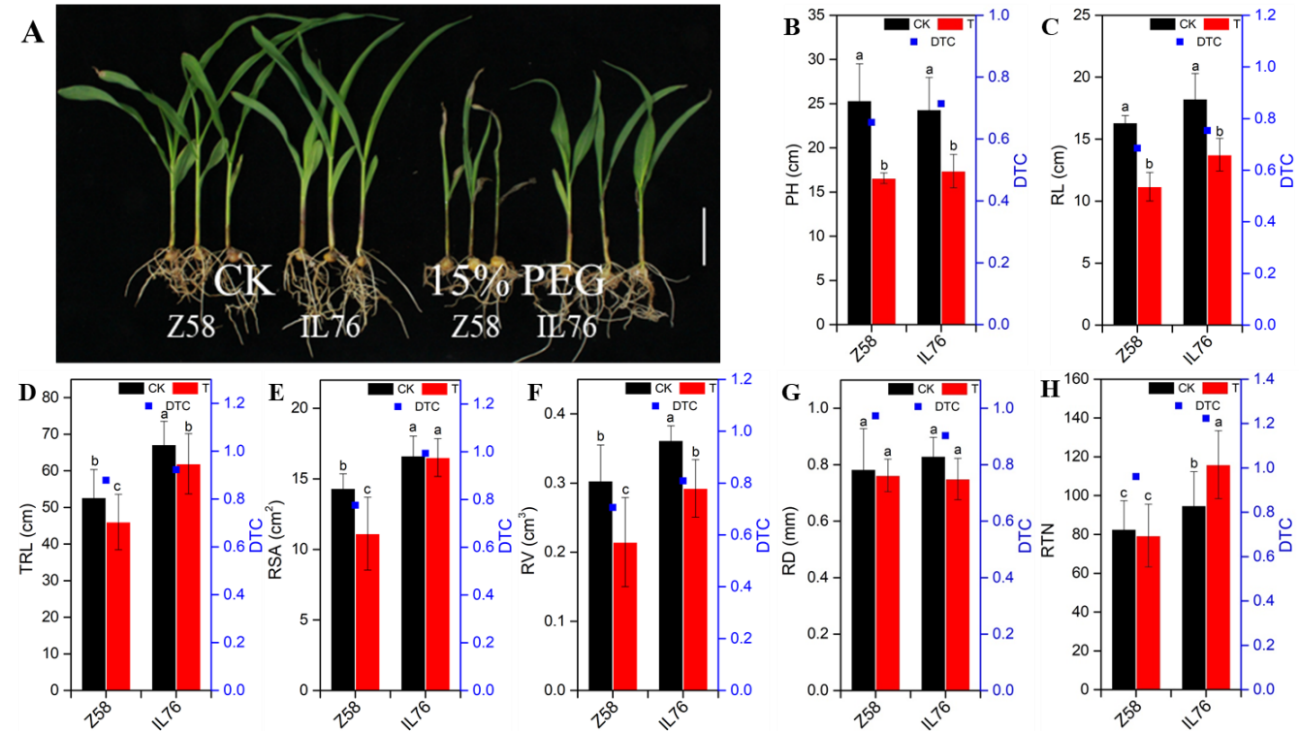


Supplementary Fig. 4 Phenotypic changes of IL76 and Z58 under normal (CK) and PEG stress (T) conditions. A shows the phenotypic changes of IL76 and Z58 under 15% PEG stress at the seedling stage for 5 days. B-H show plant height (PH), root length (RL), total root length (TRL), root surface area (RSA), root volume (RV), root diameter (RD), and root tip number (RTN), respectively. DTC in the figure is the drought tolerance coefficient, the calculation method is identical to that of STC.


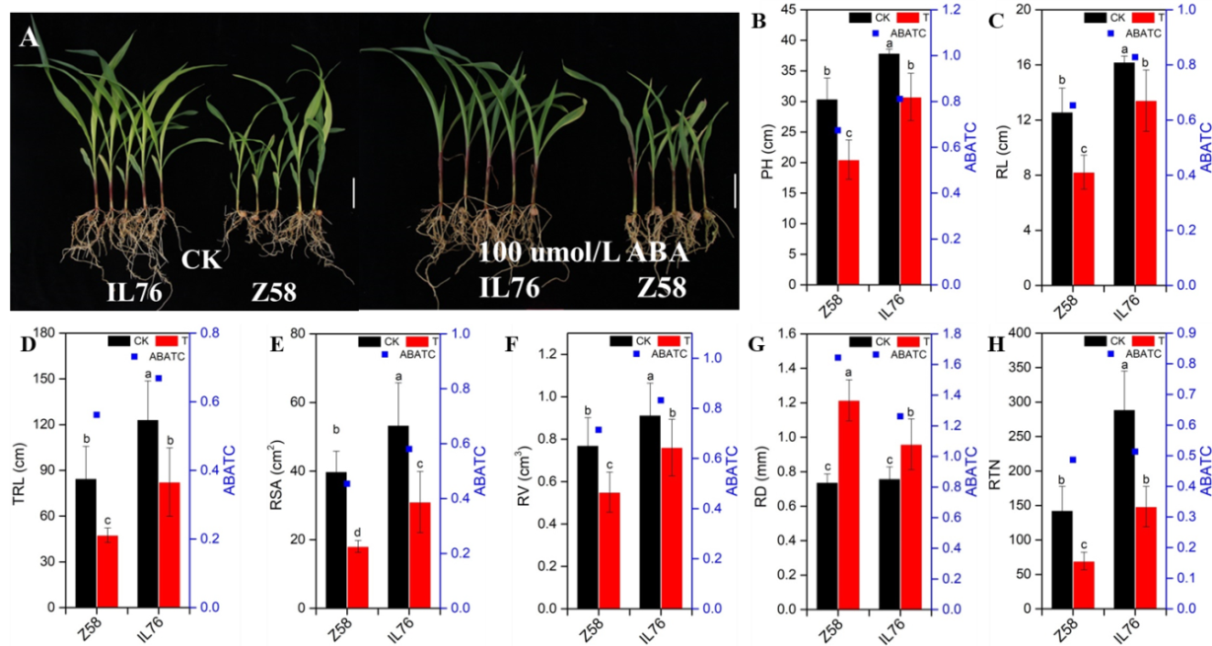


Supplementary Fig. 5 Phenotypic changes of IL76 and Z58 under ABA stress. A shows the phenotypic changes of IL76 and Z58 under 100 μmol/L ABA stress at the seedling stage for 5 days. B-H represent plant height (PH), root length (RL), total root length (TRL), root surface area (RSA), root volume (RV), root diameter (RD), and root tip number (RTN), respectively. The blue letter ABATC represents the ABA tolerance coefficient, and the calculation method is identical to that of STC.


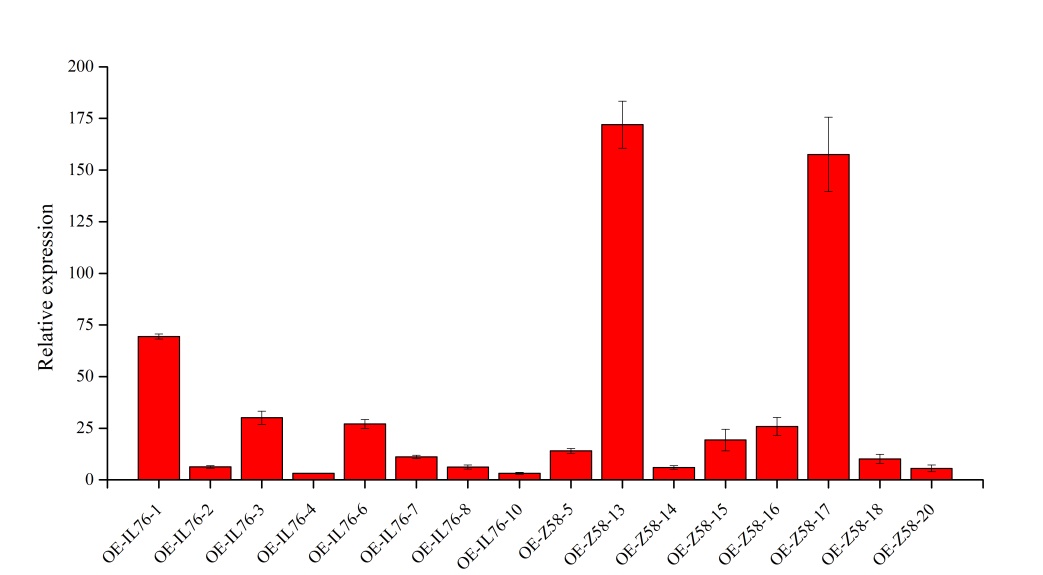


Supplementary Fig. 6 Relative expression level in leaves of *ZmSC* overexpression lines


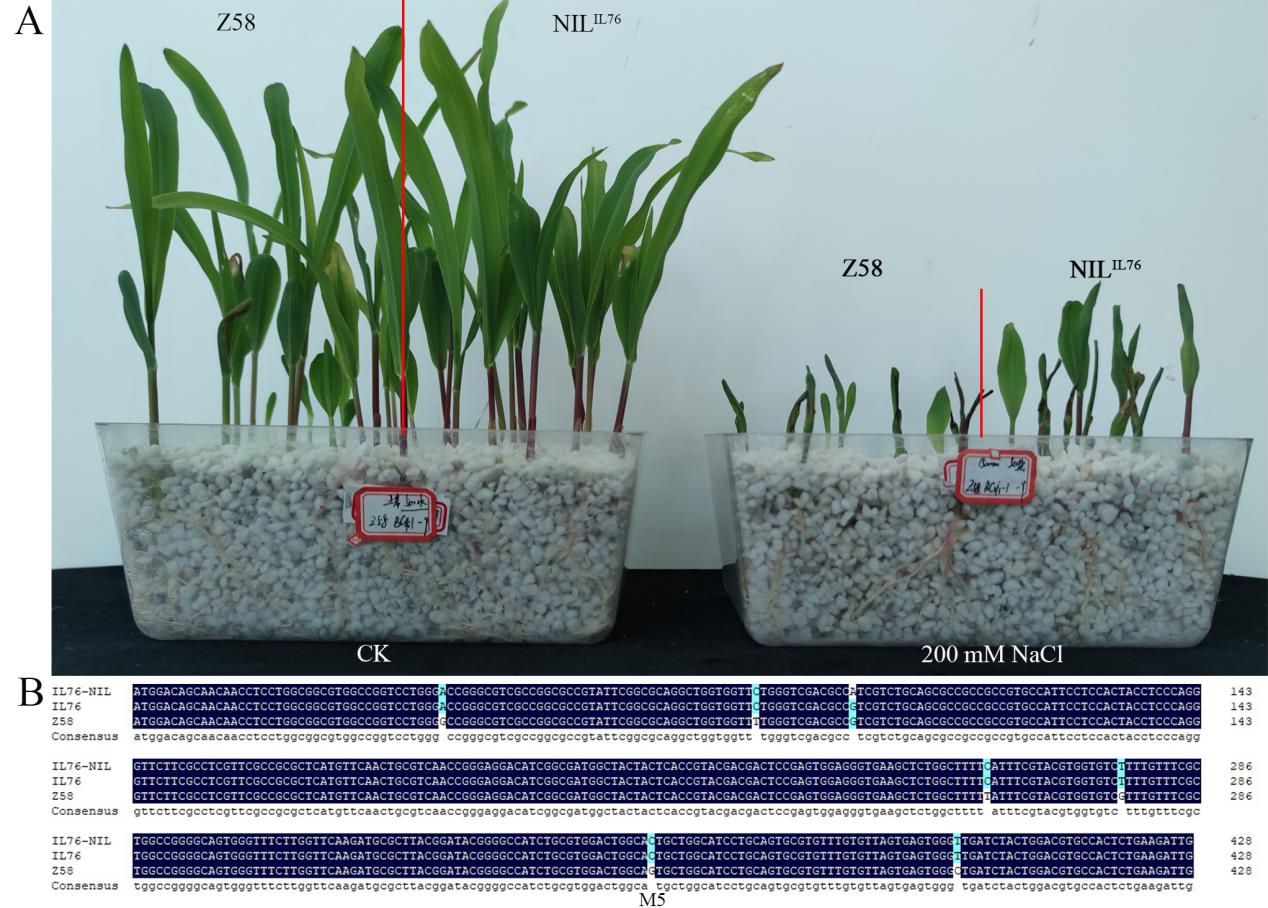


Supplementary Fig. 7 Identification of salt tolerance and mutant site detection in NIL^IL76^. A shows the phenotypic changes of Z58 and NIL^IL76^ under normal conditions and 200 mM NaCl stress for 10 days. B shows the mutant site M5.


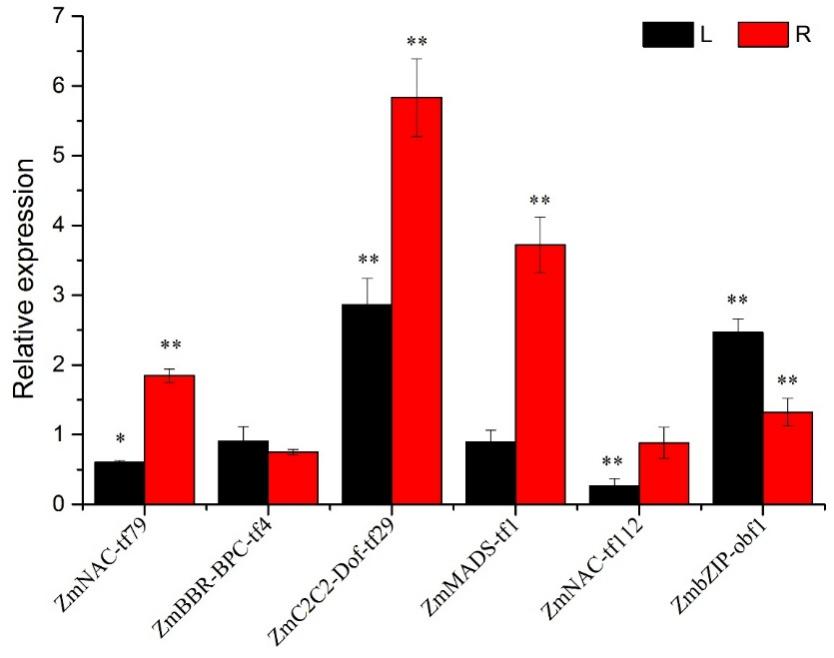


Supplementary Fig. 8 Expression of *ZmSC* promoter-interacting transcription factors in Z58 under salt stress


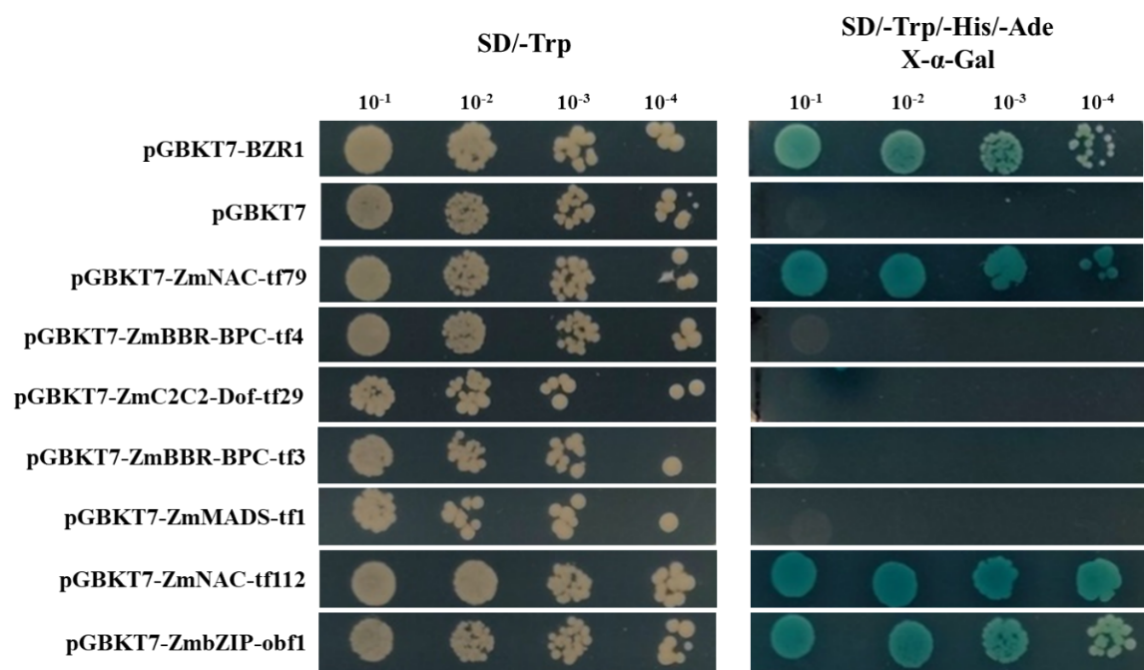


Supplementary Fig. 9 Validation of transcriptional activation activity of potential transcription factors upstream of the promoter of *ZmSC*


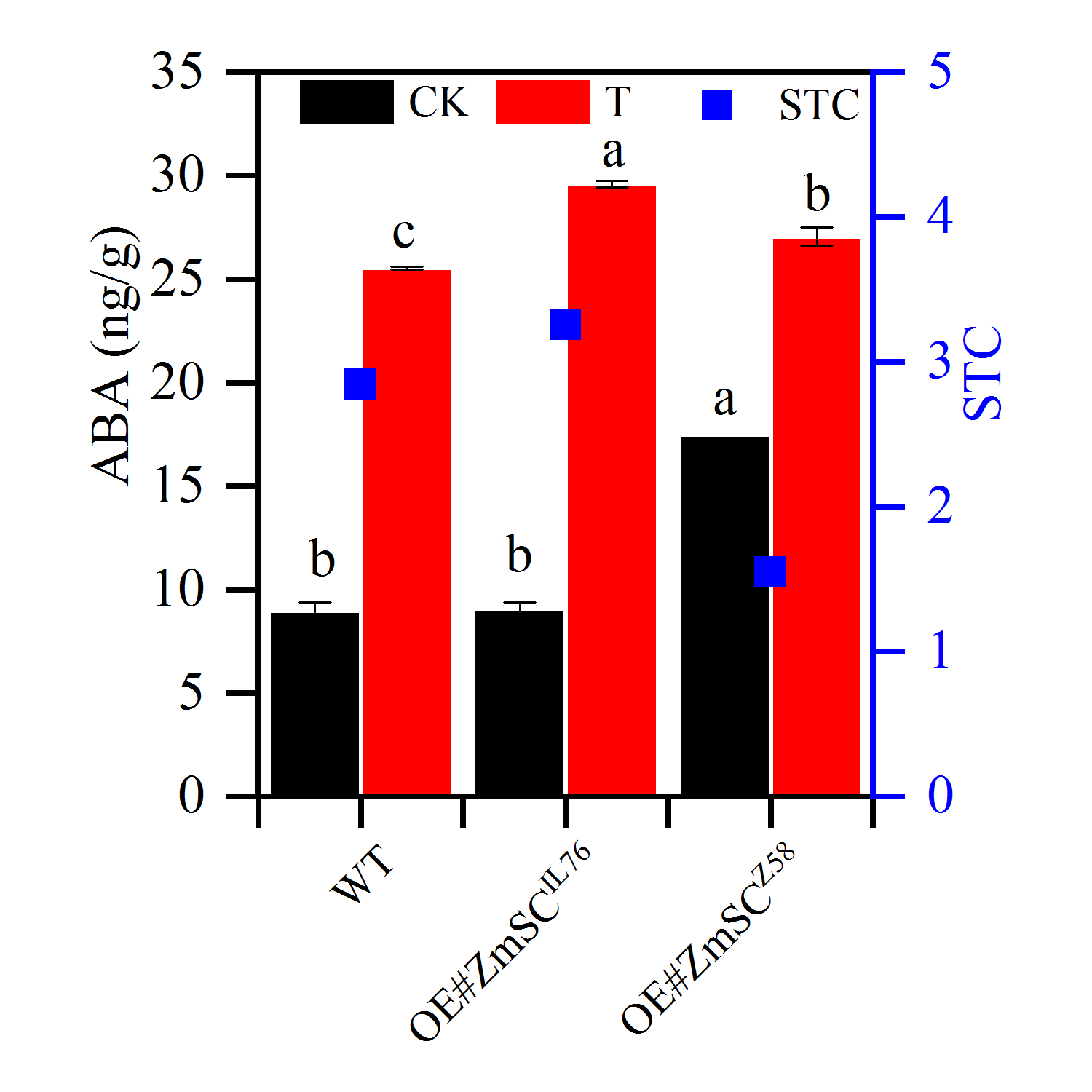


Supplementary Fig. 10 Comparative analysis of the content of ABA in overexpressing *Arabidopsis* *thaliana* under normal (CK) and salt stress (T) conditions.

Supplementary Table 1 Analysis of cis-acting elements in the promoter of *ZmSC*

| Element | Source plant | Site | Sequence | Function |  |
| --- | --- | --- | --- | --- | --- |
| ABRE | *Arabidopsis thaliana*  *Arabidopsis thaliana*  *Oryza sativa* | 193+  1565+  437- | ACGTG  ACGTG  GCCGCGTGGC | Involved in the abscisic acid responsiveness |  |
| AE-box | *Arabidopsis thaliana* | 346+ | AGAAACTT | Part of a module for light response |  |
| ARE | *Zea mays*  *Zea mays* | 791+  1472- | AAACCA  AAACCA | cis-acting regulatory element essential for the anaerobic induction |  |
| CAT-box | *Arabidopsis thaliana* | 212- | GCCACT | Related to meristem expression |  |
| CGTCA-motif | *Hordeum vulgare*  *Hordeum vulgare*  *Hordeum vulgare* | 274+  1226+  886- | CGTCA  CGTCA  CGTCA | Involved in the MeJA-responsiveness |  |
| G-Box | *Pisum sativum* | 1564- | CACGTT | Involved in light responsiveness |  |
|  | *Zea mays* | 192- | CACGTT |  |  |
| MBS | *Arabidopsis thaliana* | 479+ | CAACTG | Involved in drought-inducibility |  |
| I-box | *Flaveria trinervia*  *Zea mays* | 846-  1116+ | CCATATCCAAT  GGATAAGGTG | part of a light responsive element | |
| MYB | *Arabidopsis thaliana* | 575- | CAACAG | Drought response elements | |
| MYC | *Arabidopsis thaliana*  *Arabidopsis thaliana* | 1009+  1640+ | CATGTG  CATTTG | Drought, ABA, Low-temperature response elements | |
| Myb | *Arabidopsis thaliana*  *Arabidopsis thaliana* | 479+  994+ | CAACTG  TAACTG | Drought response elements | |
|  | *Nicotiana tabacum* | 575- | CAACAG |  |  |
| O2-site | *Zea mays* | 230+ | GATGACATGG | Involved in zein metabolism regulation | |
| CAAT-box | *Pisum sativum*  *Pisum sativum*  *Arabidopsis thaliana Brassica juncea*  *Pisum sativum*  *Pisum sativum*  *Pisum sativum*  *Pisum sativum*  *Pisum sativum* | 755-  891-  1205+  1285+  1287+  1431-  1641-  1773-  1786- | CAAAT  CAAAT  CCAAT  TGCAAATCT  CAAAT  CAAAT  CAAAT  CAAAT  CAAAT | common cis-acting element in promoter and enhancer regions | |
| TATA-box | *Oryza sativa*  *Arabidopsis thaliana*  *Arabidopsis thaliana*  *Arabidopsis thaliana Helianthus annuus*  *Brassica oleracea*  *Brassica napus* | 590+  987+  985+  989+  983-  988+  986+ | TACATAAA  TATATA  TATATA  TATA  TATACA  ATATAA  ATATAT | core promoter element around -30 of transcription start | |
| TATC-box | *Oryza sativa* | 1278+ | TATCCCA | Involved in gibberellin responsiveness | |
| TCA-element | *Brassica oleracea* | 705+ | TCAGAAGAGG | Involved in salicylic acid responsiveness | |
| TCCC-motif | *Spinacia oleracea* | 386+ | TCTCCCT | part of a light responsive element | |
| TGACG-motif | *Hordeum vulgare*  *Hordeum vulgare*  *Hordeum vulgare* | 274-  1226-  886+ | TGACG  TGACG  TGACG | Involved in the MeJA-responsiveness | |
| W box | *Arabidopsis thaliana*  *Arabidopsis thaliana* | 787-  1575+ | TTGACC | MYC2 identifies and binds elements | |
| as-1 | *Arabidopsis thaliana*  *Arabidopsis thaliana*  *Arabidopsis thaliana* | 274-  1226-  886- | TGACG  TGACG  TGACG | Involved in the MeJA-responsiveness | |
| GC-motif | *Oryza sativa* | 1051- | CGCCGCGCA | Involved in anoxic specific inducibility | |

Note: "+" forward sequence; "-" reverse sequence, the same below.

Supplementary Table 2 Transcription factors upstream of *ZmSC* gene

| TF | Gene description | Site | Sequence | P value |
| --- | --- | --- | --- | --- |
| AC205574.3_FG006 | TCP-tf4 | 264-273- | CTGGGCCCAC | 9.25e^-6^ |
| AC233865.1_FG003 | NAC-tf86 | 318-334+ | AACTTGAGAGACAAGAA | 1.69e^-6^ |
| AC233943.1_FG002 | LBD family protein | 1054-1074- | TCCGCAGTCTTCAGCGCCGCG | 5.47e^-6^ |
| GRMZM2G003944 | TCP-tf2 | 263-272- | TGGGCCCACA | 2.01e^-6^ |
| GRMZM2G004531 | NAC-tf79 | 320-338- | TTCTTTCTTGTCTCTCAAG | 1.38e^-6^ |
| GRMZM2G008374 | NAC-tf24 | 318-335- | TTTCTTGTCTCTCAAGTT | 4.38e^-6^ |
| GRMZM2G047448 | WOX family protein | 1821-1831+ | TCATTCATTCA | 3.11e^-7^ |
| GRMZM2G058518 | NAC-tf82 | 318-332- | CTTGTCTCTCAAGTT | 3.88e^-6^ |
| GRMZM2G082709 | NACtf43 | 318-332+ | ATACGGCGCCGGCGA | 2.46e^-6^ |
| GRMZM2G087804 | G2-like family protein | 838-847+ | AAGAATCTGA | 8.68e^-6^ |
| GRMZM2G118690 | BBR-BPC-tf4 | 328-348- | CTGTTTCTTTTTCTTTCTTGT | 6.53e^-6^ |
| GRMZM2G139700 | NAC-tf84 | 320-338+ | CTTGAGAGACAAGAAAGAA | 3.71e^-6^ |
| GRMZM2G140694 | C2C2-Dof-tf29 | 328-348+ | ACAAGAAAGAAAAAGAAACAG | 1.56e^-6^ |
| GRMZM2G144744 | GRAS family protein | 330-349+  332-351+ | AAGAAAGAAAAAGAAACAGA  GAAAGAAAAAGAAACAGAAA | 4.85e^-6^  8.66e^-6^ |
| GRMZM2G164735 | BBR-BPC-tf3 | 330-353+ | AAGAAAGAAAAAGAAACAGAAACT | 1.5e^-6^ |
| GRMZM2G166721 | NAC-tf16 | 318-332+ | AACTTGAGAGACAAG | 4.19e^-6^ |
| GRMZM2G171365 | MIKC_MADS-tf1 | 1346-1366+  1349-1369+  1351-1371+  322-342-  323-343-  329-349-  331-351- | GCCTGCCTTGTTTTTTTTTTT  TGCCTTGTTTTTTTTTTTTAC  CCTTGTTTTTTTTTTTTACCC  CTTTTTCTTTCTTGTCTCTCA  TCTTTTTCTTTCTTGTCTCTC  TCTGTTTCTTTTTCTTTCTTG  TTTCTGTTTCTTTTTCTTTCT | 3.06e^-6^  8.61e^-6^  5.24e^-6^  4.22e^-6^  3.46e^-6^  4.13e^-6^  2.84e^-6^ |
| GRMZM2G179885 | NAC-tf132 | 320-336+ | CTTGAGAGACAAGAAAG | 1.98e^-6^ |
| GRMZM2G361611 | bZIP-tf84 | 187-201- | AGACACGTCGGCTTC | 7.24e^-6^ |
| GRMZM2G406204 | NAC-tf78 | 320-335+ | CTTGAGAGACAAGAAA | 6.68e^-6^ |
| GRMZM2G430522 | NAC-tf114 | 319-336+ | ACTTGAGAGACAAGAAAG | 1.7e^-6^ |
| GRMZM2G444748 | bZIP-tf160 | 187-201+ | GAAGCCGACGTGTCT | 6.95e^-6^ |
| GRMZM2G456568 | NAC-tf112 | 315-335+ | GGGAACTTGAGAGACAAGAAA | 2.58e^-6^ |
| GRMZM2G479885 | bZIP-obf1 | 187-201+ | GAAGCCGACGTGTCT | 6.77e^-6^ |

Supplementary Table 3 Primers related to *ZmSC* used in this study

| Primer name | Primer sequence | Tm (℃)  Temperature | Product（bp） | Annotations |
| --- | --- | --- | --- | --- |
| M5-F | GCCTAAATTTGCAACAACTAACCT | 54.4 | 250 | Marker of introgression site M5 |
| M5-R | CATAGATACATTTCGTCGCCAAAC | 56.2 |  |  |
| ZmSC-Q 2K-F | GGCTGCCTCATCCATCCA | 57.18 | 2132 | Promoter (+1500 bp) |
| ZmSC-Q 2K-R | CGGTGACCTTCCCCGACT | 59.46 |  |  |
| ZmSC CDS-F | GGTCTCTGTCTCTCTGAAGGG | 59.5 | 560 | CDS |
| ZmSC CDS-R | AAAAGCAGTCTCCATCCTCCTC | 57.7 |  |  |
| ZmSC qPCR-F4 | GACTCCGAGTGGAGGGTGA | 59.5 | 300 | RT-qPCR |
| ZmSC qPCR-F4 | CAGAGTGGCACGTCCAGTAG | 59.5 |  |  |
| GAPDH-F | ACTTCGGCATTGTTGAGG | 59.0 | 350 | Reference gene in maize |
| GAPDH-R | AAGTCGGTAGAAACCAGAT | 58.6 |  |  |
| IPP2-F | GTATGAGTTGCTTCTCCAGCAAAG | 57.8 | 300 | Reference gene in Arabidopsis |
| IPP2-R | GAGGATGGCTGCAACAAGTGT | 57.6 |  |  |
| p2300-2F | ggtacccggggatcctctagaATGGACAGCAACAACCTCCTGG | 59.5 | 500 | Subcellular localization |
| p2300-2R | ggtactagtgtcgactctagaATCTTCAGAGTGGCACGTCCA | 58.5 |  |  |
| pREP1-F | GCGTCGACGTCTATGGACAGCAACAACCTCCTGG | 58.0 | 500 | Transformation of fission yeast |
| pREP1-R | CGGGATCCCGTCAATCTTCAGAGTGGCACGTC | 59.0 |  |  |

Supplementary Table 4 Primers related to Yeast one-hybrid assays used in this study

| Primer name | Primer sequence | Annotations |
| --- | --- | --- |
| ZmNAC-tf79-F | ATGGAGACCCCGCCGCGG | Candidate gene cloning |
| ZmNAC-tf79-R | TCACGTAGAAGAGGATTTCCATAAAC |  |
| ZmBBR-BPC-tf4-F | CGCTGCTGACAGTGTGATCC |  |
| ZmBBR-BPC-tf4-R | CGCTGCTGACAGTGTGATCC |  |
| ZmC2C2-Dof-tf29-F | CGTTTGGCTAGGGTACTCGG |  |
| ZmC2C2-Dof-tf29-R | TGAAATGACGAGCGGCAAAC |  |
| ZmBBR-BPC-tf3-F | GAATGCGGAGATGGGCTGAT |  |
| ZmBBR-BPC-tf3-R | AAGCATTGATCTTGATGCCCT |  |
| ZmMADS-tf1-F | CTCCGCCCCAATTCGGATAA |  |
| ZmMADS-tf1-R | CTAGCTTGCGTTGATTGGGC |  |
| ZmNAC-tf112-F | GGCACAACTAGTCACCCCAT |  |
| ZmNAC-tf112-R | TGCAACATCGGAACAGTCCA |  |
| ZmbZIP-obf1-F | TTTCGAGGTAAGCTAGGCCG |  |
| ZmbZIP-obf1-R | GTTGTTGAGAGGCAGCCAAT |  |
| ZmNAC-tf79-BK-F | atggccatggaggccgaattcATGGAGACCCCGCCGCGG | Transcription activation activity validation |
| ZmNAC-tf79-BK-R | tcgacggatccccgggaattcTCACGTAGAAGAGGATTTCCATAAAC |  |
| ZmBBR-BPC-tf4-BK-F | atggccatggaggccgaattcATGGACAACCTTGGGCATAGAG |  |
| ZmBBR-BPC-tf4-BK-R | tcgacggatccccgggaattcCTACCGGATGGTGATGTACCG |  |
| ZmC2C2-Dof-tf29-BK-F | atggccatggaggccgaattcATGATGGCCGGGGCACCG |  |
| ZmC2C2-Dof-tf29-BK-R | tcgacggatccccgggaattcTTAGATGGCCGCCGACGA |  |
| ZmBBR-BPC-tf3-BK-F | atggccatggaggccgaattcATGGACGACGACGACGGC |  |
| ZmBBR-BPC-tf3-BK-R | tcgacggatccccgggaattcTTATCTGATCGTTACAAACTTATTTGTACC |  |
| ZmMADS-tf1-BK-F | atggccatggaggccgaattcATGGTGCGGGGCAAGACG |  |
| ZmMADS-tf1-BK-R | tcgacggatccccgggaattcCTAGCCTGACCTGACCGCC |  |
| ZmNAC-tf112-BK-F | atggccatggaggccgaattcATGGCGCAAACTAGCCTGC |  |
| ZmNAC-tf112-BK-R | tcgacggatccccgggaattcTCACGGGAAAGGGATGGTC |  |
| ZmbZIP-obf1-BK-F | atggccatggaggccgaattcATGTCGTCGTCGTCGCTGTC |  |
| ZmbZIP-obf1-BK-R | tcgacggatccccgggaattcTCAGTAGTGGAGCATGTGCGG |  |
| ZmSC-QHISEcoRI-F | gactcactatagggcgaattcTGAAGAAAGGCTCTAAGGCTGTC | Interaction verification |
| ZmSC-QHISEcoRI-R | gcgtgagctccccgggaattcCGGCTCGCGCCCGCGGTG |  |
| ZmNAC-tf79-REC2-F | gccatggaggccagtgaattcATGGAGACCCCGCCGCGG |  |
| ZmNAC-tf79-REC2-R | accactgcttgggtggaattcTCACGTAGAAGAGGATTTCCATAAAC |  |
| ZmBBR-BPC-tf4-REC2-F | gccatggaggccagtgaattcATGGACAACCTTGGGCATAGAG |  |
| ZmBBR-BPC-tf4-REC2-R | accactgcttgggtggaattcCTACCGGATGGTGATGTACCG |  |
| ZmC2C2-Dof-tf29-REC2-F | gccatggaggccagtgaattcATGATGGCCGGGGCACCG |  |
| ZmC2C2-Dof-tf29-REC2-R | accactgcttgggtggaattcTTAGATGGCCGCCGACGA |  |
| ZmBBR-BPC-tf3-REC2-F | gccatggaggccagtgaattcATGGACGACGACGACGGC |  |
| ZmBBR-BPC-tf3-REC2-R | accactgcttgggtggaattcTTATCTGATCGTTACAAACTTATTTGTACC |  |
| ZmMADS-tf1-REC2-F | gccatggaggccagtgaattcATGGTGCGGGGCAAGACG |  |
| ZmMADS-tf1-REC2-R | accactgcttgggtggaattcCTAGCCTGACCTGACCGCC |  |
| ZmNAC-tf112-REC2-F | gccatggaggccagtgaattcATGGCGCAAACTAGCCTGC |  |
| ZmNAC-tf112-REC2-R | accactgcttgggtggaattcTCACGGGAAAGGGATGGTC |  |
| ZmbZIP-obf1-REC2-F | gccatggaggccagtgaattcATGTCGTCGTCGTCGCTGTC |  |
| ZmbZIP-obf1-REC2-R | accactgcttgggtggaattcTCAGTAGTGGAGCATGTGCGG |  |
| ZmNAC-tf79-D-F | TGCAGTTCCAGTGCGATGAT | RT-qPCR |
| ZmNAC-tf79-D--R | GATTACCAACCCGAGCACCA |  |
| ZmBBR-BPC-tf4-D-F | GTCATGGTGATGGATGTTCT |  |
| ZmBBR-BPC-tf4-D-R | CCTTTCTCGGATGGCACTGT |  |
| ZmC2C2-Dof-tf29-D-F | CTGGACTGGTACAGCGAGAC |  |
| ZmC2C2-Dof-tf29-D-R | ACCTGTGAGGATCGGGTCAT |  |
| ZmMADS-tf1-D-F | AGGAGCAAGTCCGTAAGCTG |  |
| ZmMADS-tf1-D-R | GCAGCCTTGTCTTTGCTTGA |  |
| ZmNAC-tf112-D-F | AAACCGTGCCACACCAAATG |  |
| ZmNAC-tf112-D-R | CCCATTCGGCTTCGTCAAAC |  |
| ZmbZIP-obf1-D-F | CATCGCGTCCCAGTACACC |  |
| ZmbZIP-obf1-D-R | TCAGTAGTGGAGCATGTGCG |  |
